# CD84 is a regulator of the immunosuppressive microenvironment in multiple myeloma

**DOI:** 10.1172/jci.insight.141683

**Published:** 2021-02-22

**Authors:** Hadas Lewinsky, Emine G. Gunes, Keren David, Lihi Radomir, Matthias P. Kramer, Bianca Pellegrino, Michal Perpinial, Jing Chen, Ting-fang He, Anthony G. Mansour, Kun-Yu Teng, Supriyo Bhattacharya, Enrico Caserta, Estelle Troadec, Peter Lee, Mingye Feng, Jonathan Keats, Amrita Krishnan, Michael Rosenzweig, Jianhua Yu, Michael A. Caligiuri, Yosef Cohen, Olga Shevetz, Shirly Becker-Herman, Flavia Pichiorri, Steven Rosen, Idit Shachar

**Affiliations:** 1 Department of Immunology, Department of Biochemistry, Weizmann Institute of Science, Rehovot, Israel.; 2 Judy and Bernard Briskin Center for Multiple Myeloma Research, City of Hope, Duarte, California, USA.; 3 Department of Hematologic Malignancies Translational Science and; 4 Department of Immuno-Oncology, Beckman Research Institute, City of Hope, Duarte, California, USA.; 5 Translational Bioinformatics, Center for Informatics, Department of Computational and Quantitative Medicine, City of Hope, Duarte, California, USA.; 6 Translational Genomics Research Institute, Phoenix, Arizona, USA.; 7 Sanz Medical Center, Laniado Medical Center, Netanya, Israel.; 8 Hematology Institute, Kaplan Medical Center, Rehovot, Israel.

**Keywords:** Hematology, Oncology, Cancer, Cancer immunotherapy, Cellular immune response

## Abstract

Multiple myeloma (MM) is characterized by an accumulation of malignant plasma cells (PCs) within the BM. The BM microenvironment supports survival of the malignant cells and is composed of cellular fractions that foster myeloma development and progression by suppression of the immune response. Despite major progress in understanding the biology and pathophysiology of MM, this disease is still incurable and requires aggressive treatment with significant side effects. CD84 is a self-binding immunoreceptor belonging to the signaling lymphocyte activation molecule (SLAM) family. Previously, we showed that CD84 bridges between chronic lymphocytic leukemia cells and their microenvironment, and it regulates T cell function. In the current study, we investigated the role of CD84 in MM. Our results show that MM cells express low levels of CD84. However, these cells secrete the cytokine macrophage migration inhibitory factor (MIF), which induces CD84 expression on cells in their microenvironment. Its activation leads to an elevation of expression of genes regulating differentiation to monocytic/granulocytic–myeloid-derived suppressor cells (M-MDSCs and G-MDSCs, respectively) and upregulation of PD-L1 expression on MDSCs, which together suppress T cell function. Downregulation of CD84 or its blocking reduce MDSC accumulation, resulting in elevated T cell activity and reduced tumor load. Our data suggest that CD84 might serve as a novel therapeutic target in MM.

## Graphical abstract

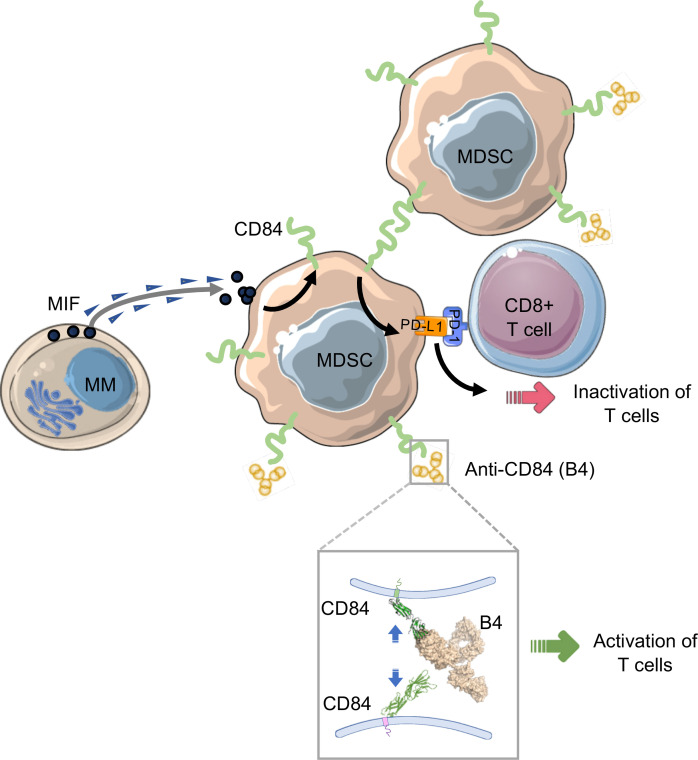

## Introduction

Multiple myeloma (MM) is a hematological malignancy caused by an accumulation of malignant plasma cells in the BM. The BM microenvironment is known to have supportive roles for MM survival, growth, and cell adhesion–mediated drug resistance. The microenvironment of the BM consists of cellular elements such as osteoblasts, stromal cells, endothelial cells, osteoclasts and immune cells, and a noncellular compartment. Interactions between myeloma cells and other cells that are part of the tumor microenvironment foster myeloma development and progression by suppression of the immune response. The interactions of MM cells with accessory cells in their microenvironment regulate the transformation of the BM microenvironment into a tumor-promoting environment through a suppressive immune milieu containing cytokines, growth factors, and chemokines (1–3).

Although the majority of studies have focused on the BM stroma and osteoclasts in MM pathogenesis, less attention has been given to the potential role of additional cell types involved in modulation of the immune response (4–6). In recent years, myeloid-derived suppressor cells (MDSC) have been shown to play a pivotal role in regulation of immune responses in cancer. MDSCs are composed of a heterogeneous myeloid cell population consisting of pathologically activated myeloid progenitors and immature myeloid cells, with robust immunosuppressive activity (7). In healthy individuals, MDSCs are rare or absent, while an increased number of MDSCs have been identified in tumors (2).

It was recently shown that MDSCs mediate suppression of T cell responses through the induction of T cell anergy and Treg development in the MM microenvironment (8), rendering lymphocytes unable to control tumor infiltration and progression (9). The immune checkpoint molecule Programmed cell death 1 (PD-1) together with its 2 ligands, PD-L1 and PD-L2, are crucial for the maintenance of a malignant prosurvival microenvironment (10). In addition, the checkpoint molecules Lymphocyte-Activation Gene 3 (LAG-3), Cytotoxic T Lymphocyte Antigen-4 (CTLA-4), 2B4, Tim-3, and CD160 play significant roles in exhaustion of T cells (11).

The signaling lymphocyte activation molecule (SLAM) family of receptors are cell surface proteins that modulate the immune response (12). CD84 (SLAMF5) is a member of the SLAM family, which self-associates, forming homophilic dimers, and is mostly expressed by the hematopoietic cells (13–16). Previous studies have shown that CD84 regulates a potentially novel survival pathway in chronic lymphocytic leukemia (CLL) (17). It bridges between CLL and various cells in the microenvironment, mediating their interaction both in vitro and in vivo (18). In addition, cell-cell interactions mediated by CD84 in humans and mice elevate the expression of PD-L1 on CLL and the surrounding microenvironment, as well as the expression of PD-1 on T cells, regulating the T cell response in the CLL microenvironment (19).

In the present study, we focused on understanding the function of CD84 in MM cancer cells and their microenvironment. Our results demonstrate that MM cells express undetectable or low levels of CD84. The malignant cells induce expression of CD84 on cells in their microenvironment by secretion of the cytokine macrophage migration inhibitory function (MIF). CD84 regulates PD-L1/PD-1 and exhaustion marker expression on MDSCs and T cells, respectively, resulting in the downregulation of the immune response. In vivo blocking of CD84 leads to reduced accumulation of MDSCs in the tumor microenvironment and to elevated activity of T cells through the control of essential functional pathways in MDSCs, leading to reduced tumor load. Our results suggest a therapeutic strategy targeting CD84 in the MM microenvironment as a promising approach to induce T cell mediated antitumor activity.

## Results

### CD84 is expressed on immunosuppressive cells in the MM microenvironment.

**Figure 1:**
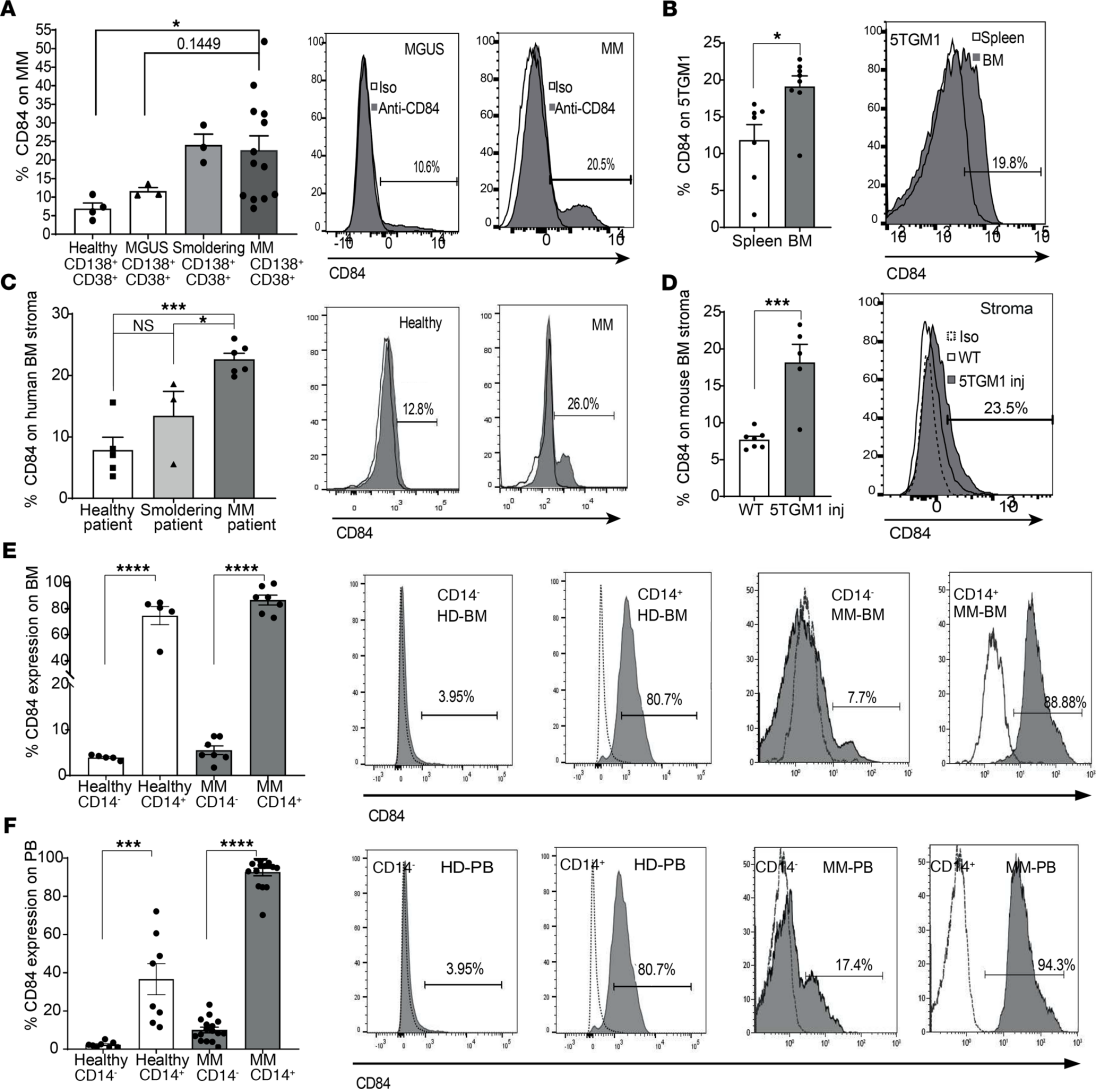
CD84 is expressed on immunosuppressive cells in the MM microenvironment. (**A**) CD84 expression was analyzed by FACS on MGUS, smoldering disease, and patient-derived CD138^+^CD38^+^ MM cells from the BM. Representative histograms, with percentages of the positively stained cells, are shown (*n* = 3–10, *P* = 0.44, 1-way ANOVA). (**B**) CD138^+^ 5TGM1 cells taken from spleen and BM of 5TGM1-injected C57BL/KaLwRij WT mice. A representative histogram demonstrating the percent of CD84^+^ BM and spleen 5TGM1 cells, is shown (*n* = 7–8). (**C**) MM BM stroma cells from MM grown in culture. Representative histograms, showing the percent of CD84^+^ cells, are shown (*n* = 3–6). (**D**) Stroma derived from BM aspirates of 5TGM1-injected C57BL/KaLwRij WT or noninjected mice grown in culture. A representative histogram, with percentages of CD84^+^ stroma cells from injected mice is shown (*n* = 3–7). (**E** and **F**) CD14^+^ cells from MM or healthy BM aspirates (**E**), or MM or healthy PB (**F**). Representative histograms, indicating percentages of positively stained cells, are shown (*n* = 7–16).

MM cells are protected from apoptosis by immunosuppressive accessory cells, which are an integral part of their microenvironment (8, 20). To determine whether CD84 plays a role in the MM disease and to further understand the interaction of these malignant cells with their microenvironment, we first evaluated CD84 expression on MM (gating is shown in Supplemental Figure 1A; supplemental material available online with this article; https://doi.org/10.1172/jci.insight.141683DS1). As shown in Figure 1A, an increase in CD84 expression was detected on MM cells compared with its expression on cells from patients with monoclonal gammopathy of unknown significance (MGUS), the precursor to MM. Analysis of the MMRF CoMMpass IA9 data set (Supplemental Figure 1B) revealed low levels of CD84 mRNA in human MM cell lines. Undetectable to low expression of CD84 was found on human MM cell lines (Supplemental Figure 1, C and D). The 5TGM1 MM plasma cell line is commonly used in a mouse model for MM (21). Analysis of CD84 expression on 5TGM1 cells derived from spleen and BM of injected mice showed significantly higher expression levels of CD84 on BM cells, compared with its expression on the malignant cells derived from the spleen (Figure 1B), suggesting that CD84 might have an important role in the BM microenvironment.

While the elevation in the expression of CD84 on malignant cells was minimal, the upregulation of CD84 expression on cells derived from the tumor microenvironment compared with its expression on healthy donors was strongly enhanced. BM stromal cells derived from MM patients expressed higher levels of CD84 compared with cells derived from both patients with smoldering disease and healthy donors (Figure 1C). In addition, analysis of BM stroma cells derived from the 5TGM1 MM murine model showed higher levels of CD84 compared with its expression on mice not carrying tumors (Figure 1D). A significant upregulation of CD84 expression was detected on BM-derived (Figure 1E) and peripheral blood–derived (PB-derived) (Figure 1F) human myeloid CD14^+^ cells (around 10 times higher) compared with its expression on CD14^–^ cells and CD14^+^ cells derived from healthy human donors, suggesting that the CD14^+^ population has a more significant role in MM patients.

**Figure 2:**
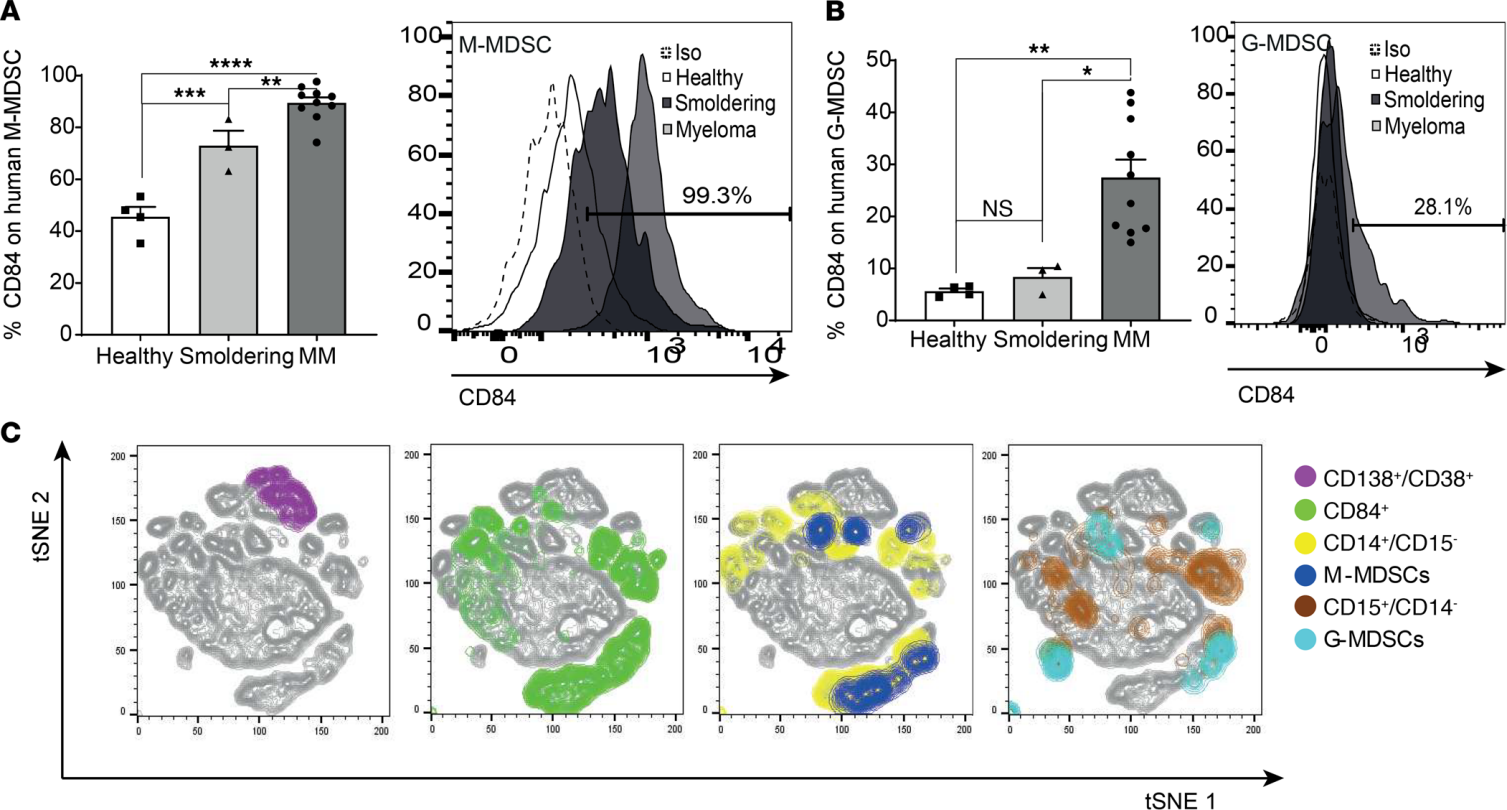
CD84 is expressed on MDSCs in the MM microenvironment. (**A**) Human BM-derived (CD14^+^, CD11B^+^, CD15^–^, HLA-DR^–^) M-MDSCs. A representative plot, showing percentages CD84^+^ MM derived M-MDSCs, is shown (*n* = 3–8). (**B**) Human BM derived (CD15^+^, CD11B^+^, CD14^–^, HLA-DR^–^) G-MDSCs. A representative histogram, and plot showing percent G-MDSCs expressing CD84, is shown (*n* = 3–8). (**C**) t-SNE plots identifying CD14^+^ cells, M-MDSC, CD15^+^ cells, G-MDSCs, MM cells, and CD84 expression. Representative t-SNE plots based on CD14^+^, CD15^+^, M-MDSC, G-MDSC, MM, and CD84^+^ cells from the MM patient BM samples (t-SNE was run with perplexity of 30 with 1000 iterations; 800,000 live cells were randomly selected). The cells are colored according to the expression level of CD14^+^/CD15^–^ for CD14; CD14^+^/CD15^–^/CD11b^+^/HLA-DR^lo^/^–^ for M-MDSC; CD15^+^/CD14^–^ for CD15; CD15^+^/CD14^–^/CD11b^+^/HLA-DR^lo^/^–^ for G-MDSC; and CD138^+^/CD38^+^ for MM cells and CD84 markers. *P < 0.05, **P < 0.01, ***P < 0.001, ****P < 0.0001, with either unpaired 2-tailed *t* test for pairwise comparisons or 1-way ANOVA with Holm-Sidak multiple corrections test for 3 groups.

Next, the MDSC populations were analyzed (gating is shown in Supplemental Figure 1E). An increased abundance of the monocytic-MDSC (M-MDSC) population and CD14^+^ cells was detected in PB derived from MM patients (Supplemental Figure 1, F–H). Since MDSCs play an important role in tumor maintenance, CD84 expression was determined on M-MDSCs (CD14^+^, CD11B^+^, HLA-DR^–^, CD15^–^) and granulocytic-MDSCs (G-MDSCs) (CD15^+^, CD11B^+^, HLA-DR^–^, CD14^–^) derived from BM patient samples. Strikingly, a significant elevation of CD84 expression was observed on both cell types derived from MM patients compared with its expression on cells derived from the earlier, premalignant stage of smoldering myeloma as well as from healthy BM (Figure 2, A and B, and Supplemental Figure 1F). To further visualize CD84 expression on the MM microenvironment, we used t-distributed stochastic neighbor embedding (t-SNE), which is a method to visualize high-dimensional data by graphing similar high-dimensional points close together and dissimilar points away from each other. As seen in Figure 2C and Supplemental Figure 2, clusters composed of M-MDSCs, G-MDSCs, and some of the MM cells expressed CD84. These findings suggest that CD84 is mainly expressed on MDSCs derived from the BM microenvironment of MM patients. The elevation of its expression suggests that CD84 might play a role in the cross talk between the tumor cells and their microenvironment.

### CD84 activation upregulates PD-L1 expression in the MM microenvironment.

**Figure 3:**
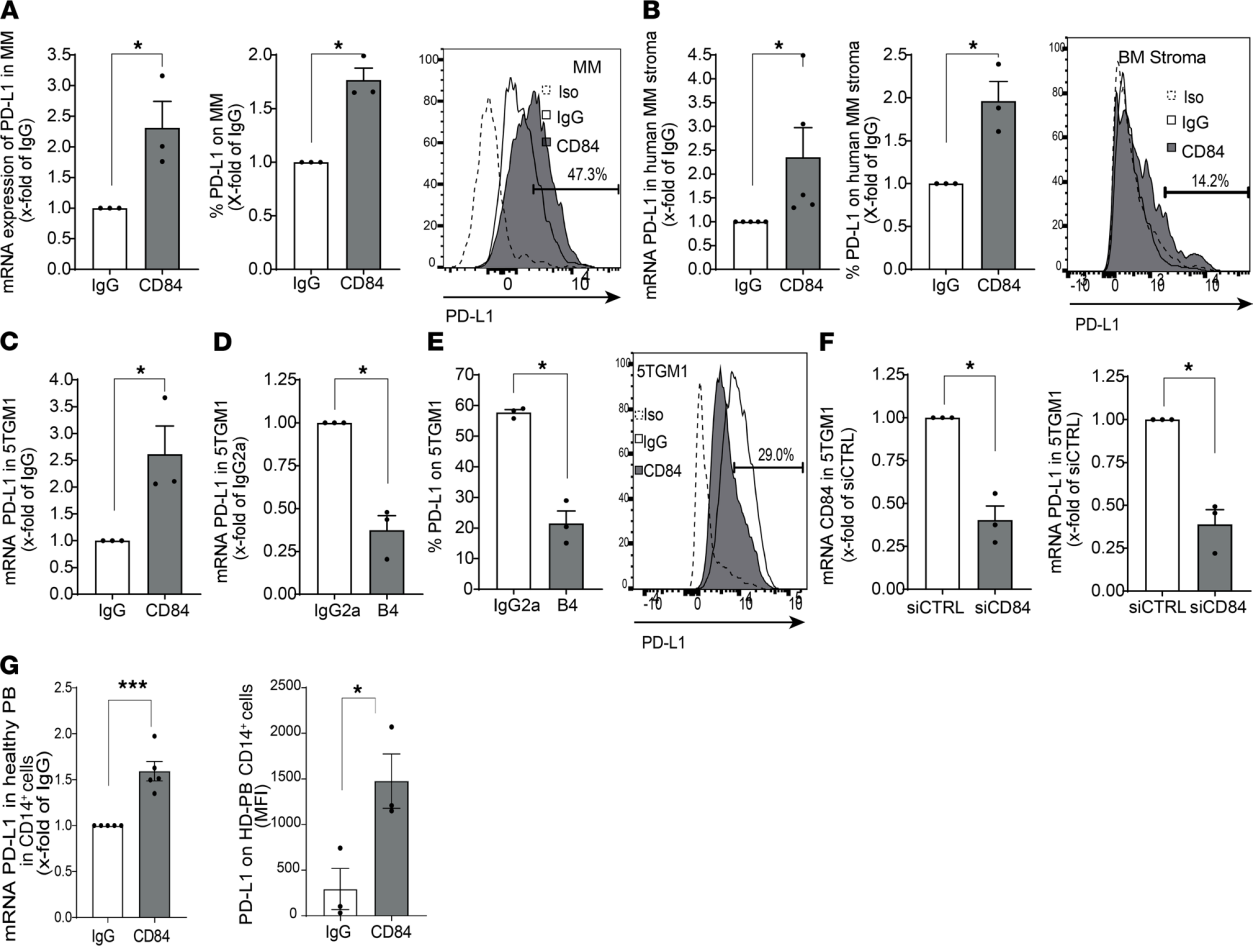
CD84 upregulates PD-L1 expression in MM cells and their microenvironment. (**A**) Sorted primary MM cells (for mRNA analysis) or total primary BM stained to identify MM cells (for protein analysis) from patients were stimulated with anti-CD84 activating or control antibodies. PD-L1 mRNA (left graph) or protein (right graph) were determined by qPCR or FACS analysis, respectively. A representative histogram, with percent of CD84^+^ cells, is shown. (**B**) BM aspirates from primary MM patients were seeded and grown until a confluent adherent layer was formed. Thereafter, CD84 was activated with anti-CD84 or control antibodies, and RNA (left graph) and protein (right graph) expression was analyzed using qPCR and flow cytometry, respectively. A representative histogram, demonstrating the percent of CD84^+^ cells, is shown (*n* = 3–5). (**C**) 5TGM1 cells were activated with anti-CD84 or control antibodies. PD-L1 message was analyzed by qPCR (*n* = 3). (**D** and **E**) 5TGM1 cells were incubated with antagonistic anti-CD84 B4 or control antibodies. PD-L1 mRNA (**D**) or protein (**E**) were analyzed by qPCR or flow cytometry, respectively. Representative histograms are shown (*n* = 3). (**F**) 5TGM1 cells were treated with either siCTRL or siCD84; RNA was purified and analyzed by qPCR for CD84 (left graph) and PD-L1 (right graph) mRNA levels (*n* = 3). (**G**) Primary PB CD14^+^ cells from healthy donors were treated with anti-CD84 activating antibody or control antibodies. PD-L1 mRNA (left graph) or protein (right graph) levels were analyzed by qPCR and flow cytometry, respectively (*n* = 3–5).

CD84 was previously shown to regulate expression of the PD-L1 immune checkpoint ligand on malignant CLL cells and in their microenvironment (19). We therefore analyzed PD-L1 expression on MM cells and cells derived from their microenvironment. Higher levels of surface expression of PD-L1 were detected on BM stromal cells of MM patients compared with its expression on cells from healthy donors (Supplemental Figure 3A), and these levels were higher in MM mouse models compared with its expression of BM stromal cells derived from tumor-free mice (Supplemental Figure 3B). To directly determine whether CD84 regulates PD-L1 expression in MM, MM BM and stromal cells taken from MM patients were stimulated with anti-CD84–activating antibody, as previously described (18, 19). PD-L1 mRNA and surface protein levels were significantly elevated in CD84-activated BM myeloma cells (Figure 3A) and stromal cells (Figure 3B) derived from the patients. In addition, activation of CD84 expression on the murine 5TGM1 cell line induced PD-L1 expression (Figure 3C), and its blocking using the B4 blocking mAb (Supplemental Figure 3C) (18, 19) lowered PD-L1 mRNA (Figure 3D) and protein (Figure 3E) levels compared with cells treated with an isotype control. Furthermore, reduced expression of CD84 in 5TGM1 cells using siCD84 resulted in downregulation of PD-L1 mRNA levels compared with the cells transfected with control siRNA (Figure 3F).

Next, the regulation of PD-L1 expression by CD84 was analyzed in CD14^+^ cells. Elevated levels of cell surface PD-L1 were detected on CD84-activated healthy donor–derived PB CD14^+^ cells (Figure 3G).

**Figure 4:**
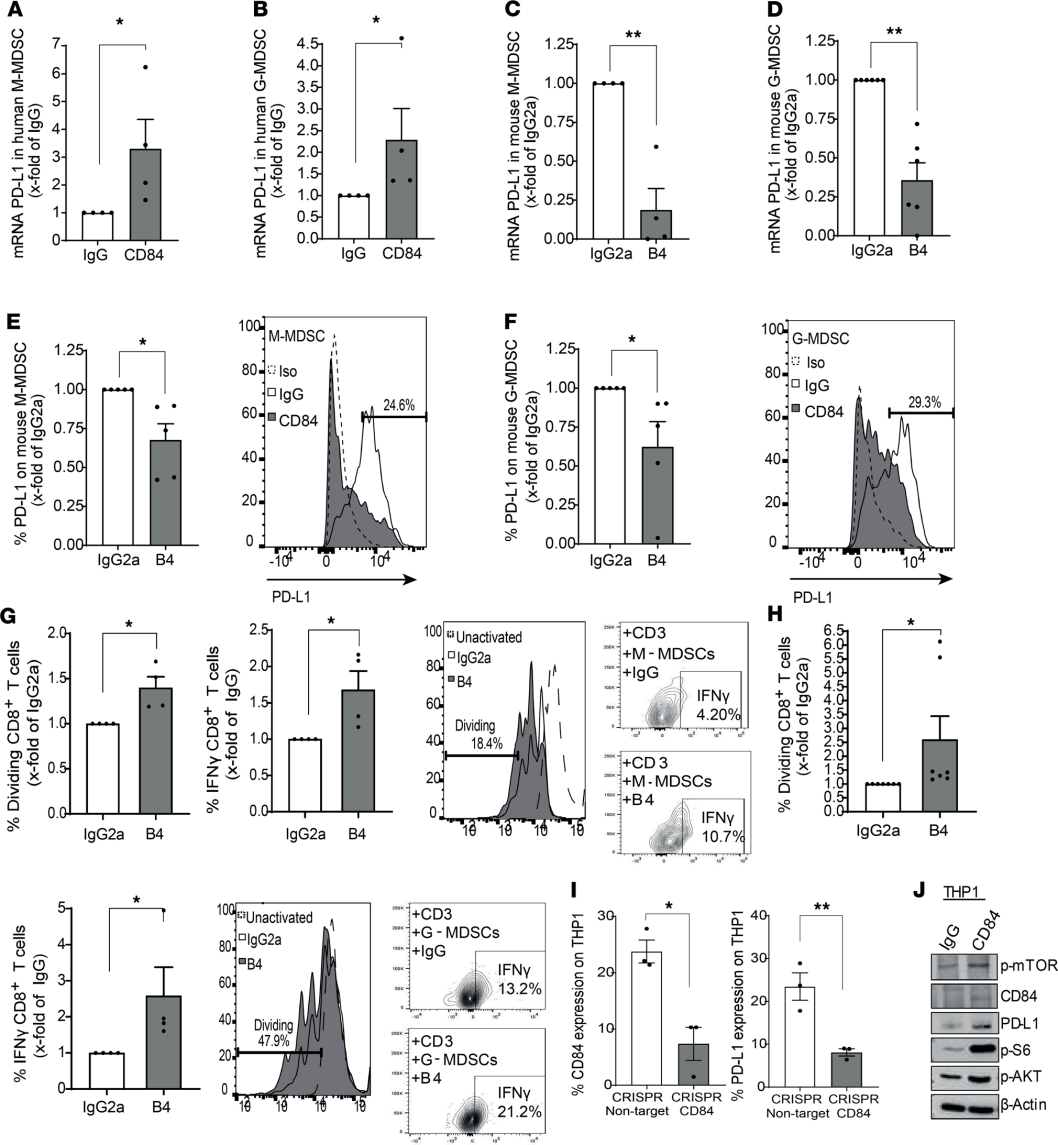
CD84 upregulates PD-L1 expression in MDSCs. (**A** and **B**) Sorted primary M-MDSCs (CD14^+^, CD15^–^, CD11b^+^, HLA-DR^–^) (**A**) and G-MDSCs (CD15^+^, CD14^–^, CD11b^+^, HLA-DR^–^) (**B**) from BM aspirates of MM samples were analyzed for PD-L1 message by qPCR. (**C**–**F**) Sorted (mRNA) or whole BM (protein) primary M-MDSCs (LY6C^+^, LY6G^–^, CD11b^+^, CD11C^–^) (**C** and **E**), and G-MDSCs (LY6G^+^, LY6C^lo^, CD11b^+^, CD11C^–^) (**D** and **F**) from BM aspirates taken from 5TGM1-injected mice were incubated in the presence of anti-CD84 (B4) blocking or isotype control antibodies and analyzed for PD-L1 mRNA (**C** and **D**) and protein (**E** and **F**) by qPCR or FACS analysis, respectively (*n* = 4). Representative histograms are shown in **E** and **F**. (**G** and **H**) Sorted M-MDSCs (**G**) and G-MDSCs (**H**) were treated with anti-CD84 B4 inhibitory or control antibody and cocultured for 72 hours with WT whole spleens at a ratio of 1:4, and the percentage of dividing CD8^+^ T cells and IFN-γ was analyzed thereafter (*n* = 4–7, *P < 0.05). (**I**) LentiCRISPR-v2 CRISPR/Cas9 system was established in the THP1 cell line with constitutive KO of CD84 expression, and protein levels of CD84 and PD-L1 were determined by flow cytometry (*n* = 3). (**J**) THP1 cells were activated with anti-CD84 or control antibodies, and CD84, p-mTOR, pAKT, pS6, and PD-L1 expression levels were determined by Western blotting analysis. *P < 0.05, **P < 0.01 with either 1-tailed (**B**, **F**, **E**, and **G**) or 2-tailed unpaired or paired 2-tailed *t* test for pairwise comparisons.

Furthermore, PD-L1 mRNA levels were elevated in CD84-activated M-MDSCs and G-MDSCs from MM patient BM aspirates (Figure 4, A and B). To confirm the effect of CD84 on PD-L1, M-MDSCs (Ly6G^–^, Ly6C^+^, CD11b^+^, CD11c^–^) and G-MDSCs (Ly6G^+^, Ly6C^lo^, CD11b^+^, CD11c^–^) from 5TGM1-injected mice were blocked using the anti-CD84 antibody B4. Following this manipulation, mRNA levels (Figure 4, C and D) as well as protein surface expression (Figure 4, E and F) of PD-L1 were significantly reduced.

Next, the effect of CD84 expressed on MDSCs was analyzed. Sorted G- or M-MDSCs from MM-injected mice were treated with B4 blocking antibody or an isotype control, incubated with T cells, and cocultured for 72 hours. As seen in Figure 4, G and H, blocking CD84 significantly reduced the suppressive function of MDSCs and increased CD8^+^ T cell division and IFN-γ secretion.

To identify the downstream events induced by CD84 that regulate PD-L1 expression, the signaling cascade induced by CD84 in THP1 cells was followed. To this end, we established a CD84 CRISPR/Cas9 system in the monocytic cell line, THP1, with constitutive KO of CD84 expression (Supplemental Figure 3D). The lack of CD84 gene expression (or CD84 deficiency) induced downregulation of PD-L1 expression on these cells (Figure 4I). Stimulating CD84 with an anti-CD84–activating antibody on CD84-expressing THP1 cells resulted in significantly elevated surface protein levels of PD-L1 compared with cells treated with IgG control antibodies (Supplemental Figure 3E). In addition, as shown in Figure 4J, levels of pAKT and pS6 whose pathways regulate PD-L1 expression (22) were markedly increased upon CD84 stimulation.

**Figure 5:**
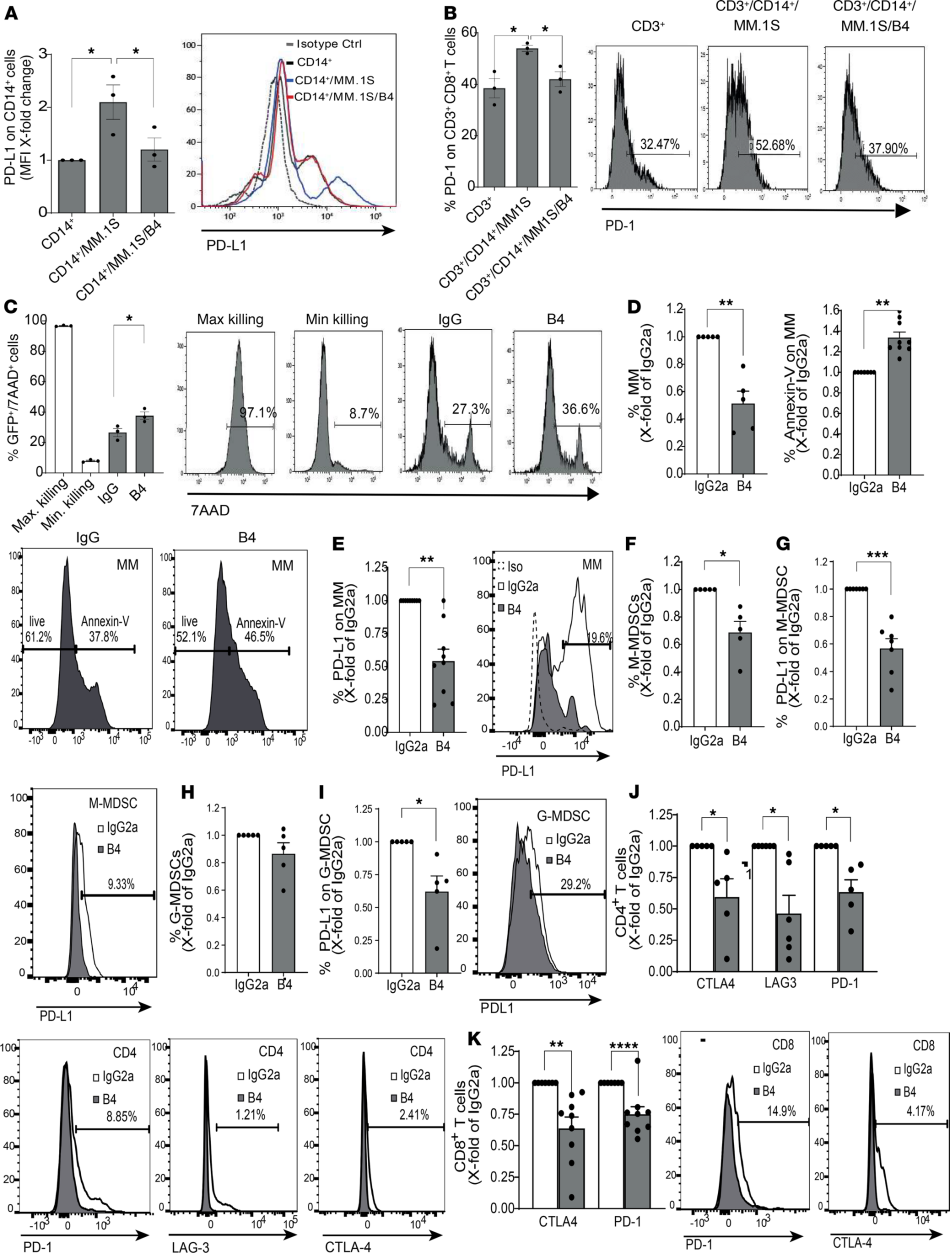
Blocking CD84 induces T cell–mediated killing of human MM cells. (**A** and **B**) PB CD14^+^ cells (**A**) or T cells (**B**) from a healthy donor were cultured alone or with MM.1S MM cell line cells at a ratio of 1:2, in the presence or absence of the anti-CD84-inhibitory (B4) or control antibodies. After 48 hours, PD-L1 (**A**) or PD-1(**B**) cell surface levels were determined by flow cytometry (*n* = 3). Representative plots are shown. (**C**) Purified PBMCs from healthy donors were treated with IgG or B4 antibodies. After 24 hours, the cells were cocultured with GFP^+^ MM.1S at a ratio of 6:1 for 16 hours. The percentage of 7AAD staining on GFP^+^ MM.1S cells was determined by flow cytometry. Representative histograms are shown (*n* = 3). (**D**–**I**) Primary MM BM aspirates were treated with B4 or IgG2a antibodies. After 48 hours, the percentage of MM cells (**D**, left graph) and annexin V staining (**D**, right graph), as well as PD-L1 expression (**E**), were determined by flow cytometry. Representative histograms, showing the percent of annexin V^+^ cells or B4-treated cells, are shown (*n* = 4–6). (**F**–**I**) M-MDSCs (**F** and **G**), and G-MDSCs (**H** and **I**) were incubated with control or B4 antibodies. Percentage of cells (**F** and **H**) and PD-L1 expression (**G** and **I**) following treatment were determined by FACS. Representative plots or histograms are shown (*n* = 4–5). (**J** and **K**) The percentage of CTLA-4, LAG-3, and PD-1 on CD4^+^ (**J**) and CD8^+^ (**K**) T cells from whole MM BM aspirates treated with B4 or IgG2a control antibodies was analyzed using flow cytometry. Representative histograms are shown (*n* = 5–7). *P < 0.05, **P < 0.01, ****P < 0.0001, with unpaired or paired *t* test for pairwise comparisons.

Next, we wished to understand whether CD84 induces an immunosuppressive microenvironment in MM patients. PB CD14^+^ cells derived from healthy donors were incubated alone or in the presence of the human MM cell line MM.1S and healthy donor T cells in the presence or absence of control IgG2a or the B4 blocking mAbs. The expression of PD-L1/PD-1 and cell survival were analyzed. The MM.1S cell line induced expression of PD-L1 on the CD14^+^ cells (Figure 5A) and PD-1 on T cells (Figure 5B). This effect was abrogated in the presence of the anti-CD84 blocking antibody B4. In addition, blocking CD84 elevated the T cell–mediated killing of MM cell lines, indicated by increased 7AAD staining of the MM cell line (MM.1S GFP^+^/Luc^+^) (Figure 5C) and increased chromium release by MM cells in a killing assay (Supplemental Figure 3H).

We further analyzed the effect of CD84 blocking on primary human MM BM aspirates from MM patients. BM aspirates were treated in culture with either control IgG2a or the B4 mAbs, and the survival of the cells was analyzed. As seen in Figure 5D, blocking CD84 induced apoptosis of the myeloma cells, leading to reduced numbers of these malignant cells. This treatment also downregulated PD-L1 surface expression on the MM cells (Figure 5E). In addition, blocking CD84 reduced the numbers of M-MDSCs (Figure 5F) and lowered PD-L1 expression on these cells (Figure 5G). This treatment had almost no effect on the number of G-MDSCs (Figure 5H), while the levels of PD-L1 were reduced on these cells, as well (Figure 5I). Furthermore, blocking CD84 reduced the expression of the T cell exhaustion markers PD-1, LAG-3, and CTLA-4 on the surface of CD4^+^ and CD8^+^ T cells (Figure 5, J and K). Taken together, these results show that CD84 regulates immunosuppression and T cell activity in MM patient samples.

### CD84 expression is elevated in a MIF-dependent manner in the MM microenvironment.

**Figure 6:**
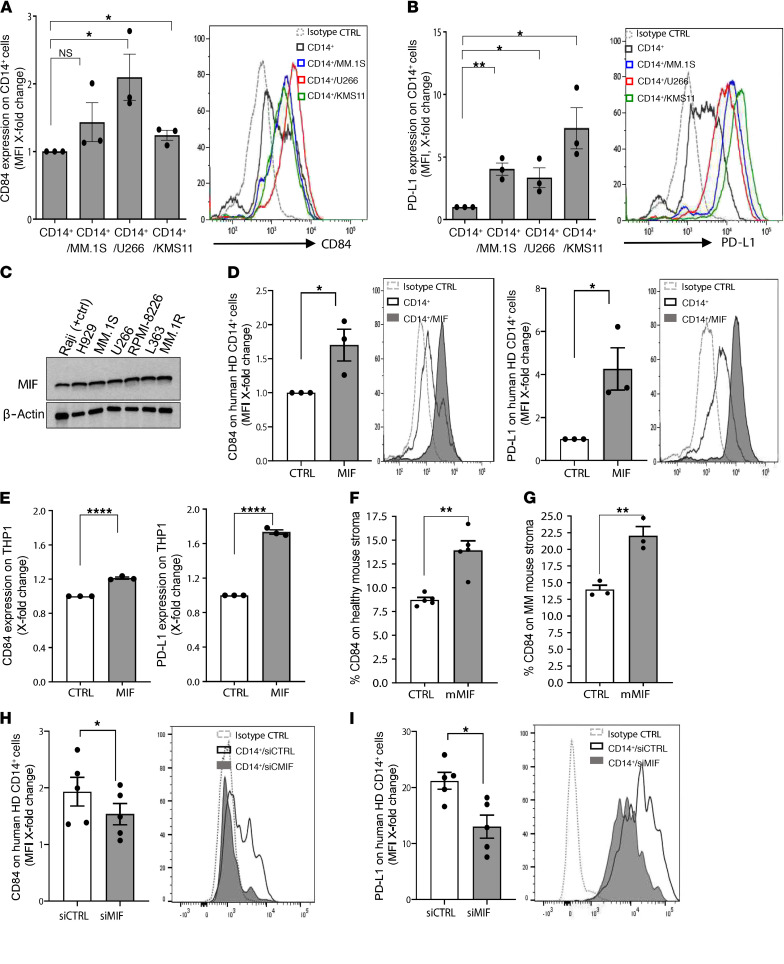
CD84 expression is regulated in a MIF-dependent manner in the MM microenvironment. (**A** and **B**) PB CD14^+^ cells derived from healthy volunteers were cultured alone or with the human myeloma cell lines (MM.1S, U266, KMS11) at a ratio of 1:2. After 48 hours, the CD14^+^ cells were analyzed by flow cytometry for CD84 (**A**) and PD-L1 (**B**) expression (*n* = 3). Representative histograms are shown. (**C**) MIF protein expression was analyzed in the human MM cell lines NCI-H929, MM.1S, U266, RPMI-8226, L363, MM.1R, and Raji cell line (positive control) by Western blot analysis. (**D** and **E**) PB CD14^+^ cells from healthy donors (**D**) or THP1 cells (**E**) were cultured with or without 200 ng/mL human recombinant MIF (hrMIF). After 48 hours, the cells were analyzed for CD84 and PD-L1 expression by flow cytometry (*n* = 3). (**F** and **G**) BM from C57BL/KaLwRij WT mice (**F**) or 5TGM1-injected C57BL/KaLwRij mice (**G**) was grown until an almost confluent adherent cell layer was formed. Thereafter, 100 ng/mL MIF was added. After 48 hours, the cells were harvested and analyzed by flow cytometry (excluding CD45^+^ and CD138^+^ cells) for CD84 expression (*n* = 3–5). (**H** and **I**) MM.1S cells were transfected using MIF or control siRNA (10 μM/mL). After 48 hours, the medium was replaced with fresh culture medium, and cells were grown an additional 72 hours for collection of supernatant. After 72 hours, the supernatants of the cells were transferred to PB CD14^+^ cells derived from healthy donors. After 48 hours, the CD14^+^ cells were harvested and analyzed by flow cytometry for CD84 (**H**) and PD-L1 (**I**) expression (*n* = 5).

MM cells express undetectable to low levels of CD84, while cells in their microenvironment express high levels of this receptor in the presence of the tumor. We therefore hypothesized that the induction by the MM cells of CD84 and PD-L1 expression on the surrounding cells may be mediated by secretion of cytokines. To determine whether myeloma cells induce CD84 and PD-L1 expression on their microenvironment, healthy donor–derived PB CD14^+^ cells were incubated alone or in the presence of 3 different human MM cell lines, MM.1S, U266, and KMS11, which do not express CD84 (Supplemental Figure 1, C and D), and PD-L1 and CD84 surface protein levels were analyzed after 48 hours. The MM cell lines induced increased cell surface levels of CD84 (Figure 6A) and PD-L1 (Figure 6B) on the CD14^+^ cells.

We have previously shown that the cytokine MIF induces CD84 expression in CLL cells by binding to its receptor CD74 (17). We therefore sought to determine whether the upregulated expression of CD84 on cells in the tumor microenvironment is modulated by MIF. MIF protein expression levels in human MM cell lines were analyzed using Western blot analysis. As demonstrated in Figure 6C, MIF was highly expressed in the human MM cell lines.

To determine whether MM cells induce CD84 and PD-L1 expression on their microenvironment in a MIF-dependent manner, PB CD14^+^ cells from 3 different healthy donors or the THP1 monocytic cell line were incubated alone or in the presence of human recombinant MIF. After 48 hours, surface protein levels of CD84 and PD-L1 were measured on these cells. MIF activation elevated expression of CD84 and PD-L1 on these cells (Figure 6, D and E).

Next, stroma taken either from healthy mice (Figure 6F) or MM-injected mice (Figure 6G) were incubated with recombinant murine MIF. MIF stimulation upregulated CD84 expression on stroma from both untreated and MM-injected mice.

To demonstrate that MIF secreted from the malignant cells regulates CD84 and PD-L1 expression on cells in the tumor microenvironment, MIF expression was knocked down in MM.1S cells. Reduction of about 40% in MIF mRNA levels was observed in cells transfected with siMIF (Supplemental Figure 3, F–G). Supernatant collected from MM.1S transfected with MIF or control siRNA was then transferred to isolated CD14^+^ PBMCs derived from a healthy donor, and CD84 and PD-L1 expression levels on the CD14^+^ cells were analyzed. Downregulation of CD84 (Figure 6H) and PD-L1 (Figure 6I) cell surface levels were detected on cells incubated with the conditioned medium containing reduced levels of MIF.

**Figure 7:**
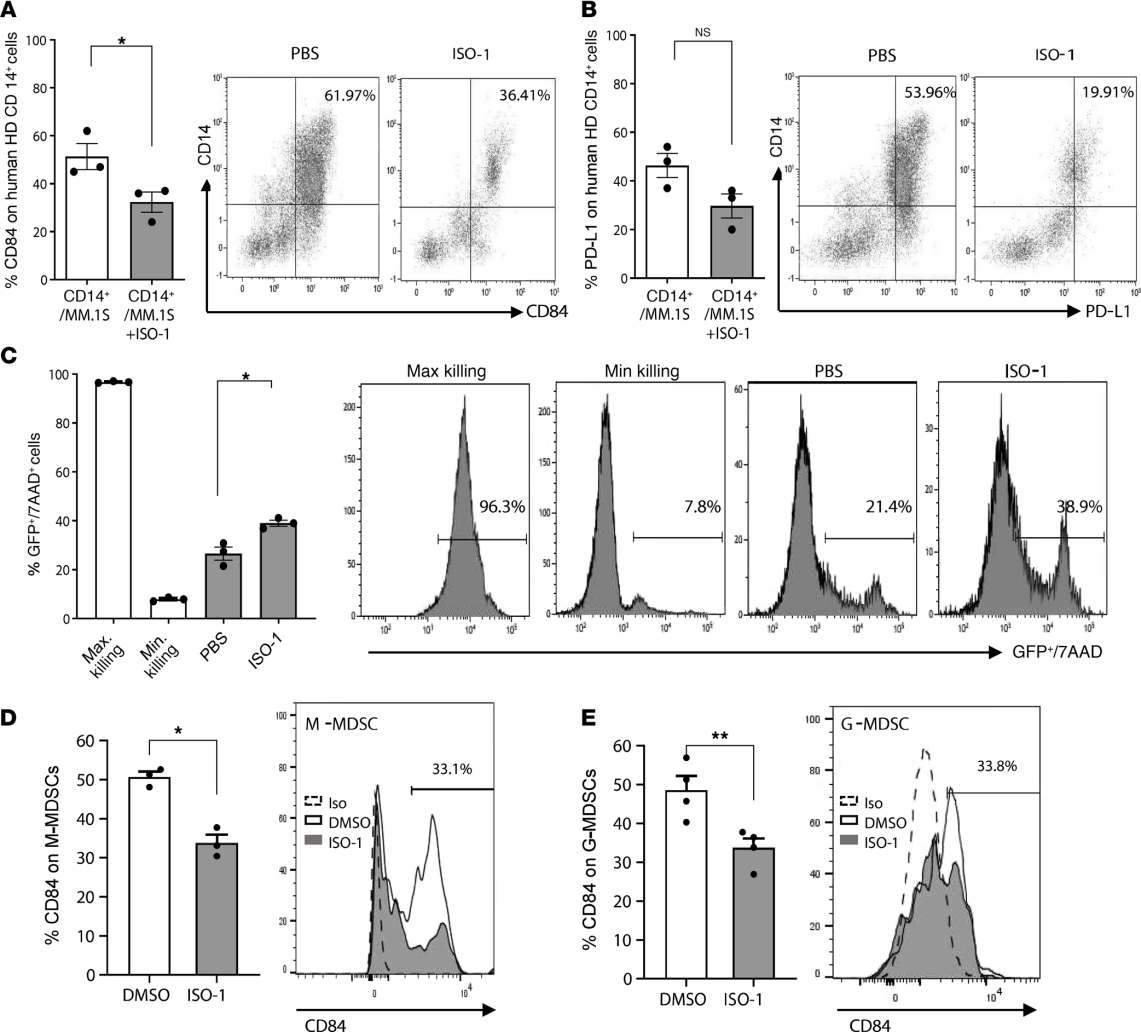
Blocking MIF reduces CD84 expression in the MM microenvironment. (**A** and **B**) PB CD14^+^ cells from healthy donors were cocultured with MM.1S at a ratio of 1:2 in presence or absence of MIF inhibitor (ISO-1). After 48 hours, CD14^+^ cells were analyzed for CD84 (**A**) and PD-L1 (**B**) expression by flow cytometry (*n* = 3). (**C**) Purified PBMCs from healthy donors were treated with or without ISO-1. After 24 hours, the cells were cocultured with GFP^+^ MM.1S at a ratio of 6:1, and the percent of 7AAD staining on GFP^+^ MM.1S cells was determined 16 hours later by flow cytometry. Representative histograms are shown (*n* = 3). (**D** and **E**) Whole BM cultures from mice bearing MM were treated with ISO-1 or control; CD84 expression was determined 48 hours later on M-MDSCs (**D**) or G-MDSCs (**E**) by flow cytometry. Representative histograms, with percentages displaying the ISO-1–treated cells, are shown (*n* = 3–4). *P < 0.05, **P < 0.01, with unpaired or paired *t* test for pairwise comparisons.

To further evaluate the effect of MIF on CD84 and PD-L1 expression on CD14^+^ cells, healthy donor–derived PB CD14^+^ cells were incubated alone or in the presence of the human MM cell line MM.1S in the presence or absence of the MIF inhibitor ISO-1. Inhibition of MIF signaling activity significantly reduced cell surface expression of CD84 (Figure 7A) and PD-L1 (Figure 7B) on CD14^+^ cells. Moreover, the PBMC obtained from healthy donors gained induced ability to kill MM1.S GFP^+^/Luc and Raji cells in the presence of ISO-1 (Figure 7C and Supplemental Figure 3, H and I). Thus, MIF regulates CD84 and PD-L1 expression levels on CD14^+^ cells.

Finally, whole BM from mice bearing MM were treated with ISO-1, and the percentage of CD84 on G- or M-MDSCs was determined. ISO-1 treatment significantly reduced the expression of CD84 on MDSCs derived from mice bearing MM (Figure 7, D and E).

Together, these results show that the expression and function of CD84 are regulated by MIF secreted from the malignant cells.

### CD84 regulates functional and immunosuppressive pathways in human MM MDSCs.

MDSCs play an important role in the support of MM cells (8). Since these cells highly express CD84 during MM progression, we next wished to follow the potential direct role of CD84 on these cells.

**Figure 8:**
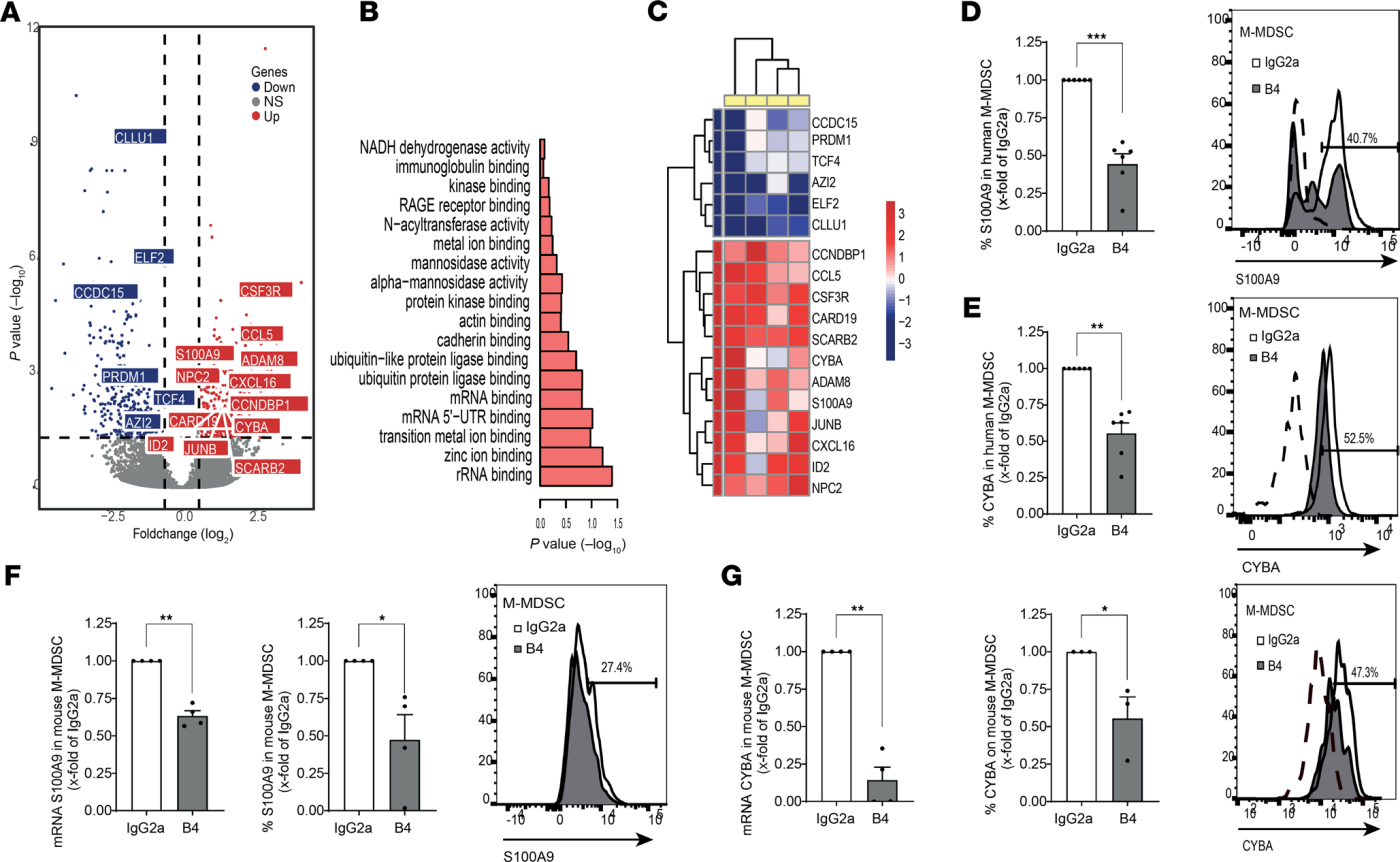
CD84 regulates functional and immunosuppressive pathways in human MM M-MDSCs. (**A**–**C**) Sorted BM M-MDSCs (CD14^+^, CD15^–^, HLA-DR^–^, CD11B^+^) were activated with anti-CD84 or -IgG1k antibodies. After 24 hours, RNA was purified and sequenced using MARS-seq. (**A**) Volcano plot for M-MDSC genes, with gray indicating non-DE genes and with DE-expressed genes shown either in blue, for downregulated, or red, for upregulated (DE genes had *P* < 0.05 after adjustment and a base mean of more than 5 after adjustment with the R package, fdrtool; *n* = 4). (**B**) Highlighted GO terms from the upregulated DE genes of M-MDSCs (Molecular Function, EnrichR). (**C**) Heatmap depicting highlighted genes from the DE genes, with each column representing the log_2_ CD84 activated to IgG control ratio value of a single patient (*n* = 4). (**D** and **E**) Purified primary MM BM samples were incubated with B4 or IgG2a antibodies. After 48 hours, M-MDSCs were analyzed for S100A9 (**D**) (*n* = 6) or CYBA (**E**) protein expression (*n* = 6). (**F** and **G**) Sorted primary M-MDSCs (message) or whole BM cultures (protein) from 5TGM1-injected mice were incubated with B4 or control antibodies. After 24 hours (mRNA, left) or 48 hours (protein, right), cells were analyzed for S100A9 (**F**) and CYBA (**G**) expression. Representative histograms are shown (*n* = 3–4). One-tailed Student’s *t* test was used for **F** and **G**.

Sorted G-MDSCs or M-MDSCs derived from patient BM aspirates (Supplemental Figure 4, A and B) were activated with either CD84 or IgG control antibodies. After 24 hours, RNA was purified, and activation was confirmed by elevation of PD-L1 expression. RNA sequencing (RNA-Seq) was conducted using MARS-seq. Principal component analysis (PCA) and dendrogram analysis showed that the samples could be identified as either CD84 activated or IgG control treatment based on similarity in gene expression profiles (Supplemental Figure 4, C–D). CD84 activated M-MDSC cells differentially expressed 433 genes (determined as genes with a *P* < 0.05 and a base mean of more than 5, after adjustment with the R package fdrtool). These genes were divided into clusters of upregulated and downregulated genes (Figure 8A and Supplemental Figure 4E). Each cluster was then analyzed for gene ontology (GO) pathways using EnrichR. The downregulated cluster did not contain pathways related to MDSC function (results not shown). However, in the upregulated genes, pathways related to MDSC function were identified (Figure 8B and Supplemental Figure 4F), including genes related to ROS production, Rage receptor binding, and abnormal T cell selection.

A more detailed analysis revealed genes including S100A9, CYBA (p22phox), and CCL5, related to the above pathways (Figure 8C). It was previously shown that, in the absence of S100A9, the MDSC population is reduced, resulting in reduced tumor size in tumor-bearing mice, while its overexpression leads to an accumulation of MDSCs and impaired DC differentiation (23). CYBA (P22phox) is a subunit of the NADPH oxidase (NOX2), and NOX2 is the main source of ROS, used by MDSCs to suppress T cells (24). Furthermore, CCL5 has previously been shown to play an important role in tumor progression by inhibiting infiltration of CD8^+^ T cells and increasing the migration of MDSCs into the tumor area (25, 26). We next validated the regulation of expression of these genes by CD84 in CD84-activated sorted human M-MDSCs from MM BM aspirates. S100A9 mRNA (Supplemental Figure 4G) and CYBA mRNA (Supplemental Figure 4H) were upregulated in CD84-activated cells. Blocking CD84 on whole cultures of primary patient MM BM reduced S100A9 (Figure 8D) and CYBA (Figure 8E) in M-MDSC cells. Moreover, blocking CD84 by the B4 mAb significantly decreased the mRNA and protein expression of S100A9 (Figure 8F) and CYBA (Figure 8G) in mouse M-MDSCs from 5TGM1-injected mice. Together, this suggests that CD84 affects pathways regulating the functionality of M-MDSCs.

**Figure 9:**
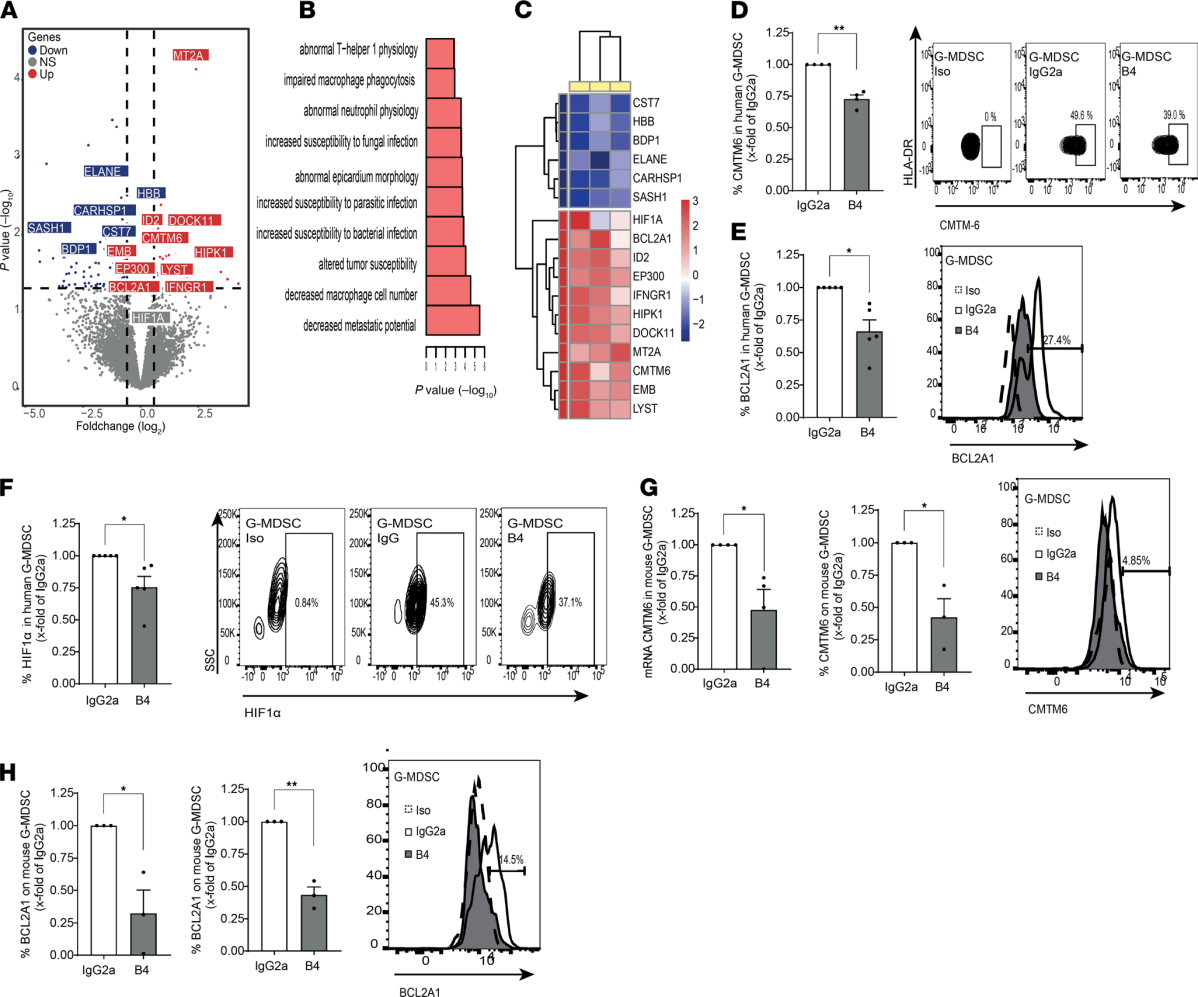
CD84 regulates functional and immunosuppressive pathways in human MM G-MDSCs. (**A**–**C**) Sorted BM G-MDSCs (CD15^+^, CD14^–^, HLA-DR^–^, CD11B^+^) were activated with anti-CD84 or -IgG1k antibodies. After 24 hours, RNA was purified and sequenced using MARS-seq. (**A**) Volcano plot for G-MDSC genes, with gray indicating non-DE genes and with DE-expressed genes shown either in blue, for downregulated, or in red, for upregulated. (**B**) Highlighted GO terms from the upregulated DE genes of G-MDSCs (MGI Mammalian Phenotype, EnrichR). (**C**) Heatmap depicting highlighted genes from the DE genes, with log_2_ CD84 activated to IgG control ratio values shown for each patient (*n* = 3). (**D**–**F**) Primary MM BM aspirates were treated with B4 or IgG2a antibodies. After 48 hours, G-MDSCs were analyzed for CMTM6 (*n* = 4) (**D**), BCL2A1 (*n* = 5) (**E**), and HIF1α (**F**) protein levels. Representative plots are shown. (**G** and **H**) Primary murine sorted G-MDSC from 5TGM1-injected mice were blocked with B4 or IgG2a antibodies. RNA was purified after 24 hours, and cells were analyzed for CMTM6 (**G**) and BCL2A1 (**H**) message and protein expression. Representative histograms are shown (*n* = 3-4). *P < 0.05, **P < 0.01, with paired *t* test for pairwise comparisons. One-tailed Student’s *t* test was used for **G** and **H**.

Activation of G-MDSC cells resulted in fewer differentially expressed genes. PCA and dendrogram analysis showed that the sequenced samples could be separated into either CD84-activated or IgG control treatment groups based on similarity in gene expression profiles (Supplemental Figure 4, I and J). These genes were divided into upregulated and downregulated clusters, and they were analyzed (Figure 9A and Supplemental Figure 4K). While the downregulated clusters were not associated with noteworthy pathways (not shown), the upregulated clustered genes were related to the HIF1α network and abnormal neutrophil physiology (Figure 9B and Supplemental Figure 4L). We then followed several specific upregulated genes — CMTM6, HIF1α (included due to upregulated pathway GO terms, although *P* = 0.18), EP300, and BCL2A1 (Figure 9C). CMTM6 is a known PD-L1 regulator on many cancer cell types (27). The transcription factor HIF1α, together with its coactivator EP300, regulates many genes involved in the hypoxia response. Previously, HIF1α has been shown to have a role both in differentiation and PD-L1 expression on MDSCs (28). Overexpressing BCL2A1 specifically on G-MDSCs was shown to increase the survival of G-MDSCs and their suppressive capabilities (29).

The expression of these genes following CD84 activation was validated by quantitative PCR (qPCR). CMTM6 (Supplemental Figure 4M), BCL2A1 (Supplemental Figure 4N), and HIF1α (Supplemental Figure 4O) mRNA levels were upregulated in sorted G-MDSCs from human MM patients. Furthermore, blocking CD84 using the B4 antibody on whole cultures of primary patient MM BM, also reduced the expression of CMTM6 (Figure 9D), BCL2A1 (Figure 9E), and HIF1α (Figure 9F). Moreover, blocking CD84 by B4 mAb reduced RNA and protein expression of CMTM6 (Figure 9G) and BCL2A1 (Figure 9H) in mouse G-MDSCs. In conclusion, CD84 regulates pathways that are important for both MDSC development and suppressive function. In addition, CD84 serves different, yet important, roles in the 2 MDSC subsets.

### The in vivo role of CD84 in MM.

**Figure 10:**
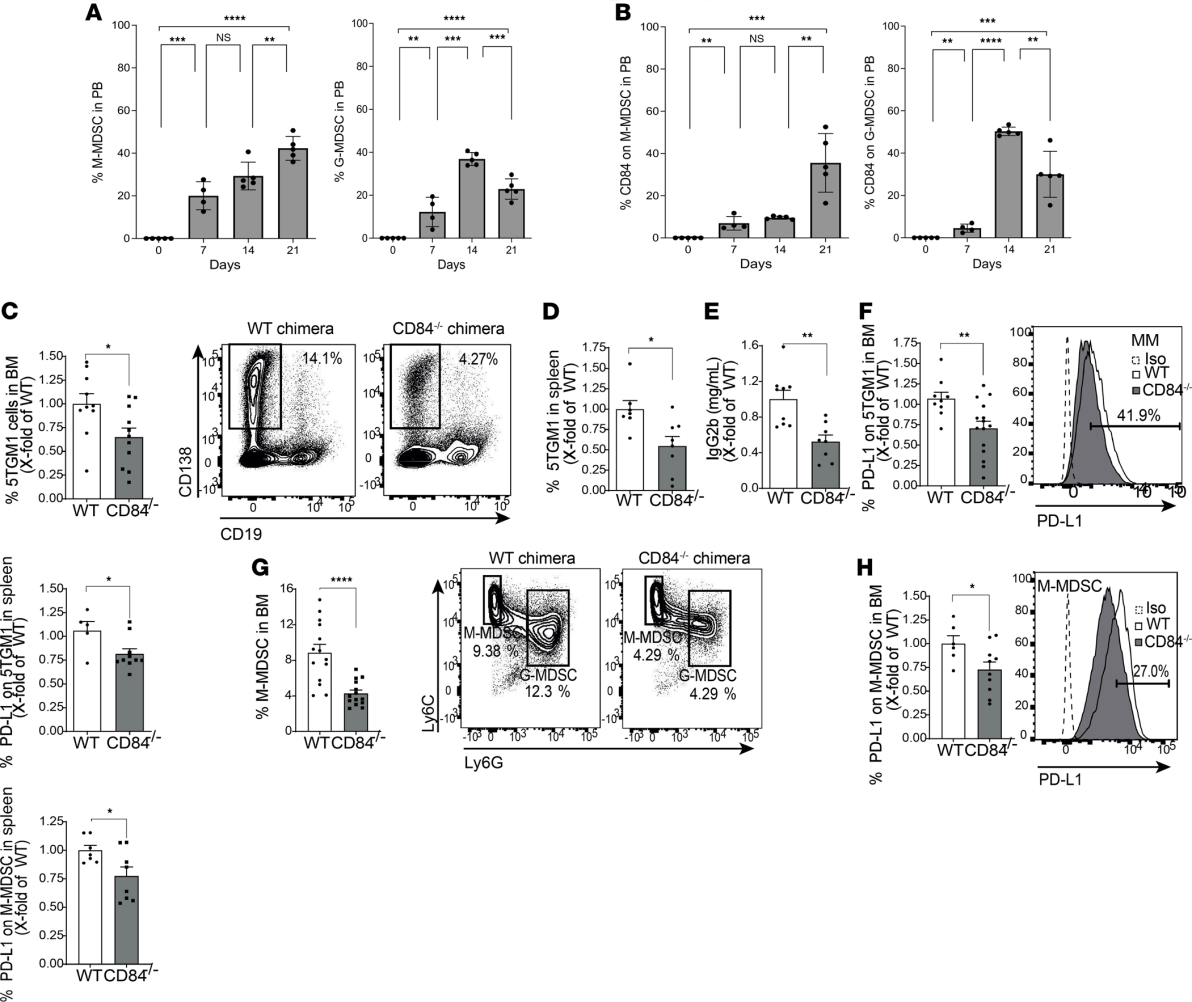
M-MDSC expansion and suppression in vivo is dependent on CD84. (**A** and **B**) 5TGM1 cells were i.v. injected into syngeneic immunocompetent C57BL/KaLwRij mice. PB samples were collected and analyzed after 1 week, 2 weeks, and 3 weeks by weekly submandibular bleeding after the injection. The percentages of M-MDSCs and G-MDSCs (**A**), and CD84 expression on M-MDSCs and G-MDSCs (**B**), were analyzed by flow cytometry (*n* = 5, **P < 0.01, ***P < 0.001, ****P < 0.0001). (**C**–**H**) WT or CD84^–/–^ mice were irradiated with 1050 Rad and injected with 1 × 10^6^ C57BL/KaLwRij BM cells after 1 day. After 60 days, the mice were injected with 1 × 10^6^ 5TGM1 cells. After an additional 21 days, the mice were sacrificed, and their BM, blood, and spleens were analyzed. (**C** and **D**) Percent of MM cells in the BM (**C**) and spleen (**D**). (**E**) IgG2b antibodies in the blood. (**F**) PD-L1 protein levels on the surface of MM from BM and spleen. Representative histogram of the BM is shown (*n* = 9–14, *P < 0.05, **P < 0.01). (**G**) Percent of M-MDSCs in the BM. Representative dot plots shown (*n* = 14, ****P < 0.0001). (**H**) PD-L1 on the surface of M-MDSCs from BM (left graph) and spleen (right graph). Representative histogram of BM is shown (*n* = 6–10, *P < 0.05).

To determine the in vivo role of CD84 in MM, 5TGM1 murine MM cells and MM.1S human myeloma cells were i.v. injected into syngeneic immunocompetent KaLwRij mice (Supplemental Figure 5A and Figure 10A) or into immunodeficient NSG mice (Supplemental Figure 5B), respectively, leading to a significant increase in MDSC numbers in the PB (Figure 10A and Supplemental Figure 5C), to increased CD84 expression (Figure 10B and Supplemental Figure 5D) on MDSC cell surface, and to increased PD-1 cell surface expression on BM CD8^+^ T cells (Supplemental Figure 5E).

To directly analyze the role of CD84 expressed on the microenvironment in vivo, chimeric mice were generated. CD84^–/–^ or WT mice were lethally irradiated and injected with KaLwRij BM cells. After 2 months, 5TGM1 cells were injected into these mice, and after an additional 3 weeks, the mice were sacrificed, and their BM, blood, and spleens were harvested and examined (Supplemental Figure 5F). Deficiency of CD84 on stroma cells resulted in a reduction in tumor load in the BM (Figure 10C) and spleen (Figure 10D), resulting in smaller spleen size (Supplemental Figure 5G) as well as reduced IgG2b in the blood (Figure 10E). PD-L1 expression on the MM cells was downregulated in both compartments (Figure 10F). Analysis of the MDSC populations revealed that mice deficient in CD84 on their stroma cells showed a significant reduction in the abundance of M-MDSCs (Figure 10G), which expressed lower levels of PD-L1 (Figure 10H).

**Figure 11:**
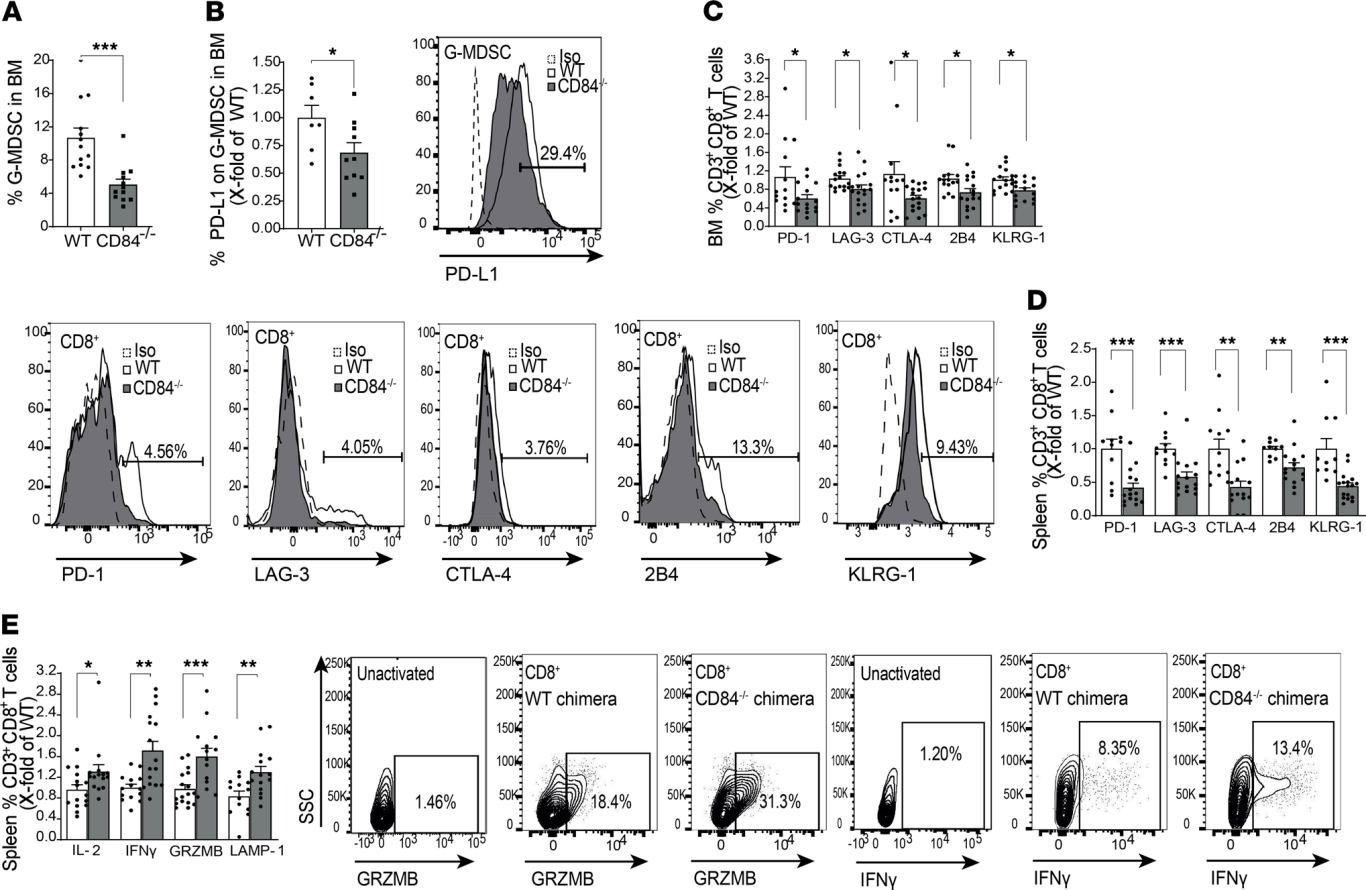
G-MDSC expansion and suppression in vivo is dependent on CD84. (**A**–**E**) WT or CD84^–/–^ mice were irradiated with 1050 Rad and injected with 1 × 10^6^ C57BL/KaLwRij BM cells after 1 day. After 60 days, the mice were injected with 1 × 10^6^ 5TGM1 cells. After an additional 21 days, the mice were sacrificed, and their BM, blood, and spleens were analyzed. (**A**) Percent of BM G-MDSCs (*n* = 12–13, ***P < 0.001). (**B**) PD-L1 expression on the surface of BM G-MDSCs. Representative histogram is shown (*n* = 7–10, *P < 0.05). (**C**–**E**) BM-derived (**C**) and spleen-derived (**D**) T cells were analyzed for PD-1, LAG-3, CTLA-4, 2B4, and KLRG-1. Representative histograms, with percentages displaying the CD84^–/–^ cells, are shown (**C**; *n* = 13–17, *P < 0.05) (**D**; *n* = 11–15, **P < 0.01, ***P < 0.001). (**E**) Splenic T cells were activated for 24 hours using anti-CD3 antibody. Brefeldin-A was added to the culture for the last 2 hours. Representative dot plots are shown (*n* = 13–17, *P < 0.05, **P < 0.01, ***P < 0.001).

Furthermore, lower levels of G-MDSCs were observed in the BM (Figure 11A). PD-L1 cell surface expression on these cells was downregulated, as well (Figure 11B). These results further emphasize the role of CD84 as a regulator of MDSC number and function. Analysis of T cells derived from the chimeric mice lacking CD84 on stroma revealed reduced levels of exhaustion markers, among them PD-1 (Figure 11C), as well as an increase in IL-2, IFN-γ, GRZMB, and LAMP-1 in the BM (Figure 11C) and spleen (Figure 11, D and E). Thus, CD84 expressed on stroma cells in the tumor microenvironment regulates immunosuppression through MDSCs and functionality of T cells, which together result in the support of MM cell viability.

**Figure 12:**
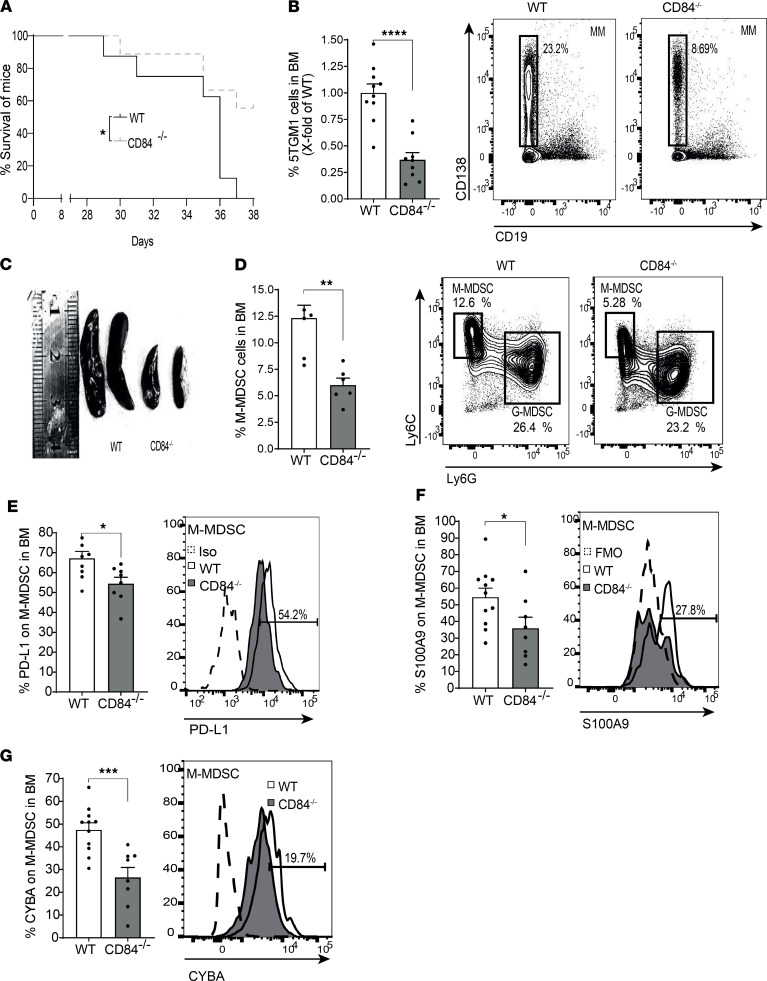
Loss of CD84 reduces M-MDSC expansion and MM tumor load. CD84^–/–^ mice backcrossed to C57BL/KaLwRij for 6–7 generations and C57BL/KaLwRij mice were injected with 5TGM1 cells. After 28 days for all experiments except the survival study, the mice were sacrificed and their BM, blood, and spleens were analyzed. (**A**) Survival curve (*n* = 8–9, *P < 0.05, log-rank test). (**B**) Percentage of MM cells in the BM (*n* = 9–10, ****P < 0.0001). Representative dot plots are shown. (**C**) Spleens derived from the WT and CD84^–/–^ mice. (**D**) Percentage of BM M-MDSCs. Representative dot plots are shown (*n* = 5–6, **P < 0.01). (**E**–**G**) PD-L1 (**E**), S100A9 (**F**), and CYBA (**G**) protein levels were determined in BM M-MDSCs by FACS. Representative histograms are shown (*n* = 5–11, *P < 0.05, ***P < 0.001).

We further analyzed the role of CD84 in MM in CD84^–/–^ mice that were backcrossed with C57BL/KaLwRij mice for 6–7 generations. 5TGM1 cells were i.v. injected to the CD84^–/–^/KaLwRij or C57BL/KaLwRij mice, and mice survival was followed. CD84 deficiency significantly enhanced the survival of the injected mice (Figure 12A). Furthermore, mice sacrificed on days 25–28 days showed a significantly reduced tumor load in the BM (Figure 12B) and spleen (Supplemental Figure 6, A and B), and their spleen size was smaller (Figure 12C). Furthermore, PD-L1 cell surface levels were reduced on MM cells in the spleen (Supplemental Figure 6C). Analysis of MDSCs in these backcrossed mice revealed a reduced number of M-MDSCs in the BM (Figure 12D), spleen (Supplemental Figure 6D), and blood (Supplemental Figure 6E). Deficiency of CD84 resulted in lower levels of PD-L1 (Figure 12E), S100A9 (Figure 12F), and CYBA (Figure 12G) compared with their expression on WT BM M-MDSCs. The expression of PD-L1 was also reduced on PB M-MDSCs lacking CD84 (Supplemental Figure 6F).

**Figure 13:**
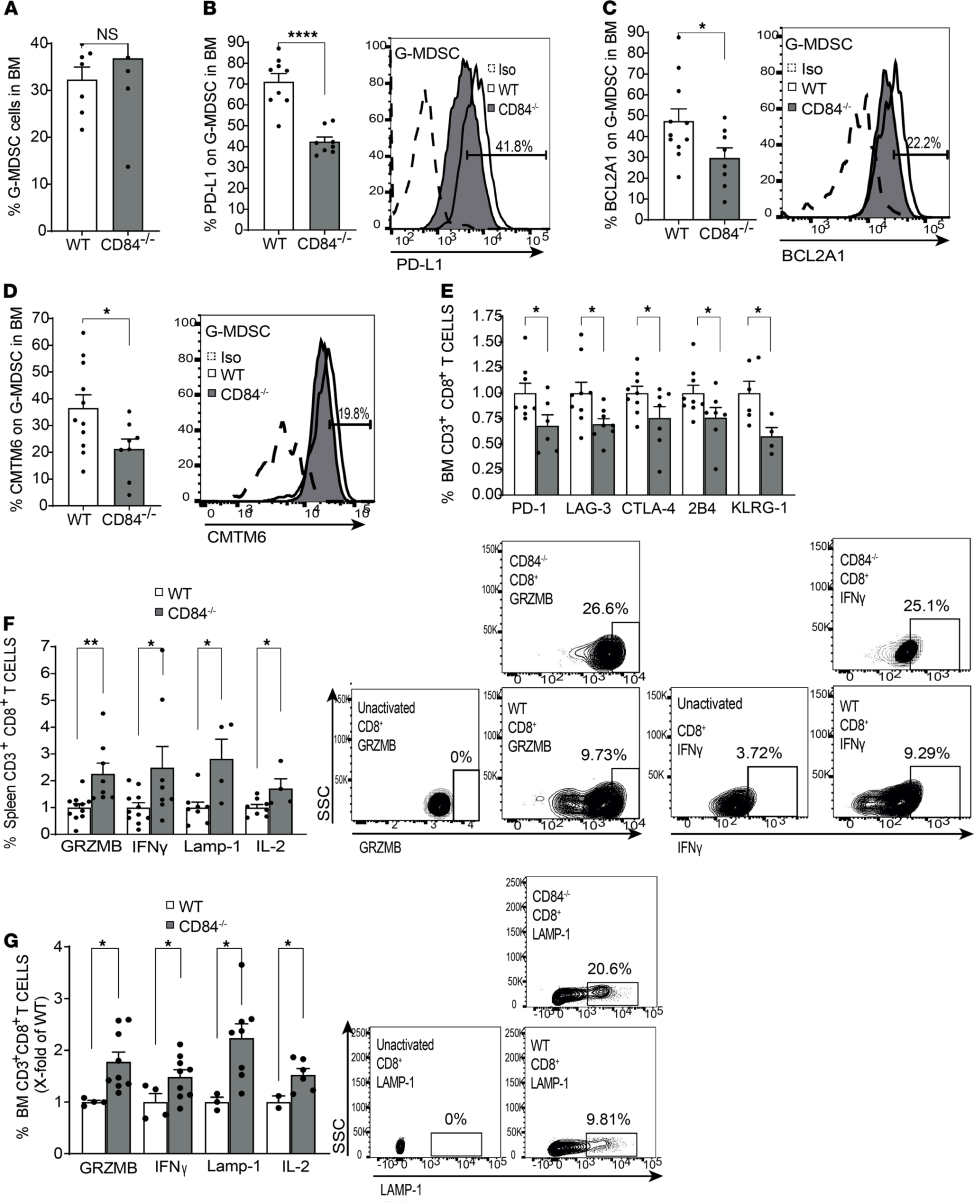
Loss of CD84 reduces G-MDSC expansion and T cell suppression. CD84^–/–^ mice backcrossed to C57BL/KaLwRij for 6–7 generations and C57BL/KaLwRij mice were injected with 5TGM1 cells. After 28 days, the mice were sacrificed and their BM, blood, and spleens were analyzed. (**A**) Percentage of BM G-MDSCs. (**B**–**D**) PD-L1 (**B**), BCL2A1 (**C**), and CMTM6 (**D**) protein levels were determined on BM G-MDSCs by FACS. Representative histograms are shown (*n* = 4–11, *P < 0.05, ****P < 0.0001). (**E**) BM-derived T cells analyzed for PD-1, LAG-3, CTLA-4, 2B4, and KLRG-1 cell surface levels by FACS (*n* = 6–9, *P < 0.05). (**F** and **G**) Splenic (**F**) or BM (**G**) T cells were activated for 24 hours with anti-CD3 in the presence of the Brefeldin-A during the last 2 hours. GRZMB, IFN-γ, LAMP-1, and IL-2 protein levels were analyzed by FACS. Representative dot plots of GRZMB and IFN-γ are shown (*n* = 4–11, *P < 0.05, **P < 0.01). One-tailed Student’s *t* test was used for **E** and **G**.

CD84-deficient G-MDSCs were reduced in the spleen and blood (Supplemental Figure 6, G and H), while no change was found in the BM (Figure 13A). The CD84-deficient BM G-MDSCs expressed significantly reduced levels of PD-L1, BCL2A1, and CMTM6 compared with WT G-MDSCs (Figure 13, B–D), and blood G-MDSCs expressed lower levels of PD-L1 on these cells (Supplemental Figure 6I). Thus, deficiency of CD84 on MDSCs reduced the number of MDSCs and their immunosuppressive functions in vivo.

Next, the effect of CD84 on BM T cells was studied. BM CD84^–/–^ T cells expressed reduced levels of the exhaustion markers PD-1, LAG-3, CTLA-1, 2B4, and KLRG-1 (Figure 13E; Supplemental Figure 6, J–L; and representative histograms in Supplemental Figure 6M), while no change was observed in the expression of these markers on splenic T cells (Supplemental Figure 6N). In addition, the CD84-deficient splenic (Figure 13F) and BM (Figure 13G) T cells elevated the expression of cytotoxic factors. Thus, CD84 deficiency in the environment of mice injected with MM results in a significant reduction in MDSC immunosuppression, resulting in a significant increase in T cell activity, and leading to a substantial reduction in tumor load.

### Therapeutic interference using the anti-CD84 blocking antibody.

**Figure 14:**
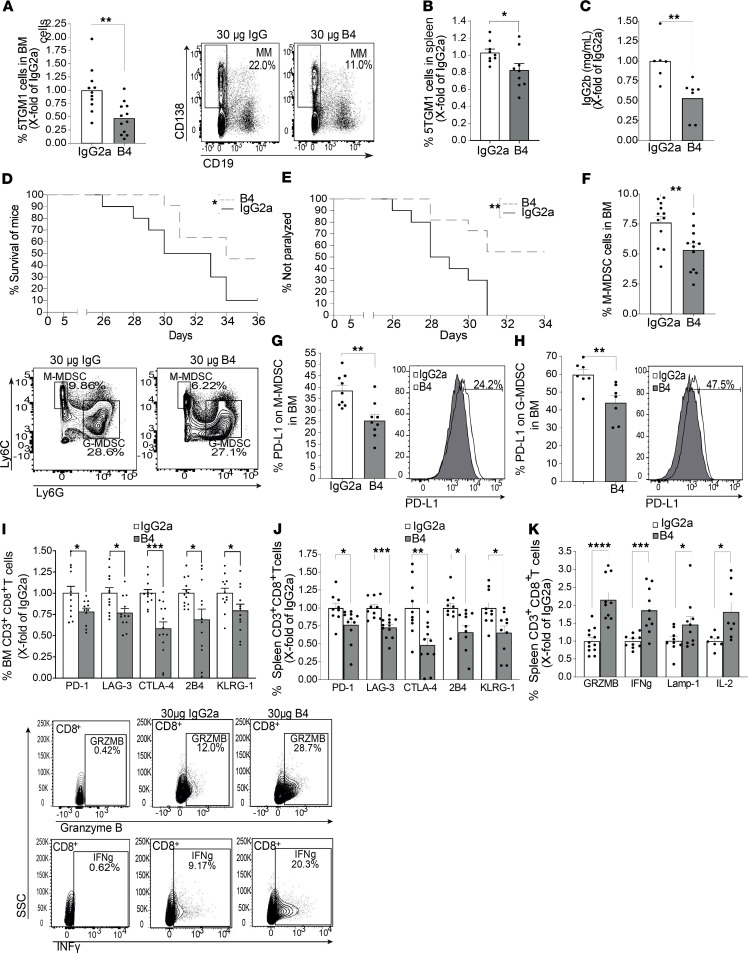
B4 has a therapeutic effect on MM-injected mice. (**A**–**K**) C57BL/KaLwRij mice were injected with 1 × 10^6^ 5TGM1 cells. After 2 weeks, the mice were divided into 2 groups and treated with 30 μg B4 anti-CD84 blocking antibody or IgG2a control. (**A**–**C**) Percent of 5TGM1 cells in the BM (**A**) and spleen (**B**) and fold of chance in serum IgG2b (**C**), following 5 treatments with B4. Representative dot plots are shown (*n* = 9–12). (**D** and **E**) Survival curve (**D**) and percent of paralyzed mice (**E**) (*n* = 10–11). (**F** and **G**) Percent of BM M-MDSCs (**F**) and PD-L1 expressed on their surface (**G**) following 5 injections. Representative dot plot is shown (*n* = 12 [**F**]; *n* = 9 [**G**]). (**H**) Percent of PD-L1 on G-MDSCs in the BM following 5 injections. Representative histogram, are shown (*n* = 7). (**I** and **J**) BM-derived (**I**), and spleen-derived (**J**) T cells were analyzed for PD-1, LAG-3, CTLA-4, 2B4, and KLRG-1 by FACS (*n* = 9–13). (**K**) Splenic T cells were activated for 24 hours with anti-CD3 together with Brefeldin-A during the last 2 hours. GRZMB, IFN-γ, LAMP-1, and IL-2 protein levels were analyzed by FACS with representative dot plots (*n* = 7–12). One-tailed Student’s *t* test was used for **K**.

To determine whether CD84 can serve as a potential target for treatment of MM, its activity was blocked in vivo by injection with the B4 anti-CD84 blocking mAb. 5TGM1 cells were injected into 2 groups of syngeneic KaLwRij mice, and 2 weeks later, the mice were injected with either IgG2a control mAb or B4 anti-CD84 (Supplemental Figure 7A). Two days after the last of the 5 injections, mice were sacrificed, and tumor load, MDSCs number, and PD-L1 expression, as well as T cell functionality, were examined. B4 mAb treatment led to a significant decrease in malignant cell numbers in the BM (Figure 14A), in spleen size and cell number (Figure 14B and Supplemental Figure 7, B and C), and in levels of IgG2b in the blood (Figure 14C), leading to increased survival of the mice (Figure 14D). Furthermore, while the isotype control mAb–injected mice were all paralyzed by day 31, 40% of mAb B4–injected mice were still able to walk (Figure 14E).

Next, the MDSC populations were analyzed. Blocking CD84 reduced the accumulation of M-MDSCs in the BM (Figure 14F) and led to decreased expression of PD-L1 on these cells in BM (Figure 14G), blood (Supplemental Figure 7D), and spleen (Supplemental Figure 7, E and F). No difference in the number of G-MDSC cells was detected in the BM (Supplemental Figure 7G); however, PD-L1 expression levels on these BM, spleen, and blood-derived cells was reduced (Figure 14H and Supplemental Figure 7, H–J).

Next, a significant elevation in the expression of T cell exhaustion markers on BM (Figure 14I) and spleen (Figure 14J and representative histograms shown in Supplemental Figure 7K) and splenic CD8 production of the cytokines IL-2 and IFN-γ — and of the markers of cytotoxicity GRZMB and LAMP-1 (Figure 14K) — was observed in B4 mAb–treated mice. No effect was observed on the number of Tregs (Supplemental Figure 7L) or PD-1 expression (Supplemental Figure 7M) on Tregs in the BM, showing that CD8^+^ cells are the primary affected T cell population.

**Figure 15:**
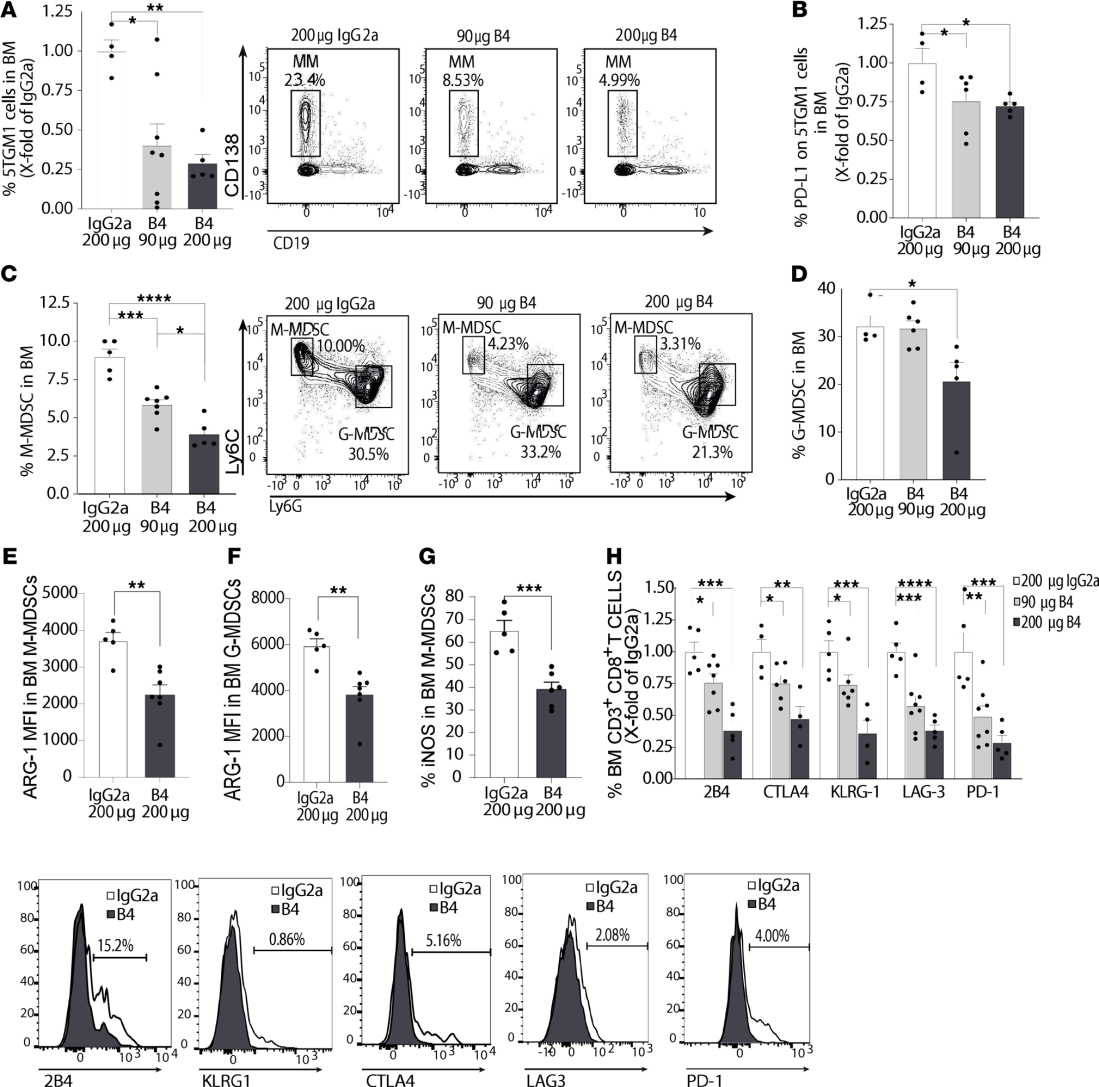
B4 reduces tumor load, MDSCs, and T cell suppression in a dose-dependent manner. (**A**–**H**) C57BL/KaLwRij mice were injected with 1 × 10^6^ 5TGM1 cells. After 2 weeks, the mice were divided into 3 groups and treated with 5 injections of 90 or 200 μg of B4, or 200 μg IgG2a. (**A**) Percentage of 5TGM1 cells in the BM following 5 treatments. Representative dot plot shown (*n* = 4–7). (**B**) Percent of PD-L1 on 5TGM1 cells in the BM following 5 treatments (*n* = 4–6). (**C**) Percent of M-MDSCs in the BM following 5 injections. Representative dot plots are shown (*n* = 4-6). (**D**) Percent of G-MDSCs in the BM following 5 injections. Representative dot plot shown in **C** (*n* = 4–6). (**E** and **F**) Arginase-1 staining on BM M-MDSCs (**E**) and G-MDSCs (**F**) following 5 injections of 200 μg B4 or 200 μg IgG2a (*n* = 5–7, **P < 0.01). (**G**) iNOS staining on BM M-MDSCs following 5 injections of 200 μg B4 or 200 μg IgG2a (*n* = 5–7, ***P < 0.001). (**H**) BM-derived T cells analyzed for PD-1, LAG-3, CTLA-4, 2B4, and KLRG-1. Representative histograms are shown (*n* = 4–8). *P < 0.05, **P < 0.01, ***P < 0.001, ****P < 0.0001, with unpaired *t* test for pairwise comparisons and 1-way ANOVA with Holm-Sidak multiple corrections test for 3 groups.

Finally, the dose-dependent effect of the B4 mAb treatment was analyzed. As seen in Figure 15A, the percentage of myeloma cells in the BM decreased by 50% in mice treated with 30 μg B4/mouse and by close to 75% in mice treated with 200 μg B4/mouse, compared with mice treated with 200 μg/mouse IgG2a control, with a similar trend found in the spleen (Supplemental Figure 8, A and B). In addition, while the lowest dose of B4 mAb did not affect PD-L1 expression on the 5TGM1 cells themselves (data not shown), the higher dose had an effect in the BM (Figure 15B) and spleen (Supplemental Figure 8C). Similarly, the abundance of BM M-MDSCs (Figure 15C), splenic M-MDSCs (Supplemental Figure 8D), BM G-MDSCs (Figure 15D), PD-L1 expression on M-MDSCs (Supplemental Figure 8E), splenic G-MDSCs (Supplemental Figure 8F), and PD-L1 expression on G-MDSCs (Supplemental Figure 7G) was also affected in a dose-dependent manner, though BM G-MDSCs only decreased relative to control at the higher dose of 200 μg B4/mouse. Moreover, to show the effect of CD84 blocking on MDSC function, Arginase-1 and iNOS expression levels were examined. MDSC-derived Arginase-1 and iNOS are known T cell suppression pathways that operate through metabolism of L-Arginine (30, 31). The higher dose of 200 μg B4/mouse reduced Arginase-1 expression in G- and M-MDSCs (Figure 15, E and F; representative histograms in Supplemental Figure 8, H and I) and iNOS in M-MDSCs (Figure 15G; representative histograms in Supplemental Figure 8J). In addition, T cells were analyzed under these conditions. All exhaustion markers decreased in response to treatment, in a dose-dependent manner (Figure 15H).

Taken together, blocking CD84, can be used to reduce the expansion of MDSCs and, thus, decrease the immunosuppressive microenvironment, resulting in increased T cell activation, which in turn leads to a decrease in tumor load.

## Discussion

MM is considered an incurable disease, despite the improved understanding of its underlying disease biology and the suggestion of potential therapies. The role of the microenvironment and immune function have been extensively studied in this disease. The main dysregulated subsets within the MM microenvironment are Tregs, Th17, DCs, and MDSCs (32). T cells display an exhausted phenotype in this malignancy, showing downregulated expression of activating receptors and upregulated expression of exhaustion markers, such as PD-1 (33, 34). Moreover, upon stimulation, T cells from MM-bearing mice express reduced levels of LAMP-1, a protein marking degranulation of cytotoxic vesicles, and secrete significantly less IFN-γ than their counterparts from healthy mice, further indicating their reduced antitumor capabilities (35).

The cell surface receptor CD84 mediates the interaction of CLL cells with their microenvironment (18) and regulates PD-L1 expression on CLL cells and their microenvironment, resulting in the regulation of T cells in the BM (19). The current study focused on the expression and function of CD84 on MM cells and the MM microenvironment. We show that the cytokine MIF released from the MM cells upregulates CD84 expression on cells in their microenvironment and that PD-L1 expression is induced on these cells. In addition, T cells derived from the tumor microenvironment express elevated levels of PD-1, which inhibits their functionality and controls their immunosuppressive behavior. Our results demonstrate that blocking CD84 reduces PD-L1 expression on cells in the MM microenvironment and reduces PD-1 expression — in addition to other T cell exhaustion markers such as 2B4, CTLA-4, KLRG-1 and LAG-3 — on T cells, resulting in elevated T cell immunity, leading to a significant decrease in tumor load both in an in vivo mouse model and in patient-derived samples.

Both M- and G-MDSCs accumulate in MM patients and affect tumor progression (36). MDSCs are classified as a heterogeneous collection of immunosuppressive cells derived from myeloid progenitors. These cells are mostly composed of granulocytic and monocytic MDSCs, and they are important components in many different types of tumor microenvironments. They support the tumor by suppressing the T cell–antitumor response, mediated by reactive oxygen species (ROS), Arginase, and nitric acid synthase (37). In addition, these cells express high levels of PD-L1 in the tumor tissue (37, 38).

Our results show an important regulatory role for CD84 in the function of MDSCs. CD84 controls the expression of S100A9 in these cells. S100A8 and S100A9 have been shown to regulate the differentiation of MDSCs at the expense of mature cells, such as DCs and macrophages. In addition, S100A8 and S100A9 also act as chemoattractants for the MDSCs (39). Furthermore, CD84 upregulates the expression of CYBA and PD-L1 on M-MDSCs in the environment of the malignant cells. The NADPH oxidase complex is composed of p40^phox^, p47^phox^, p67^phox^, gp91^phox^ and CYBA (p22^phox^), which generate ROS (40). The expression of these subunits is upregulated in M-MDSCs isolated from non–small cell lung cancer patients controlling the immune suppression of T cells mediated by ROS (41). In addition, the elevated differentiation of MDSCs induced by S100A9 has been attributed to ROS and NADPH oxidase, thereby linking CYBA and S100A9 (42) as essential pathways for M-MDSC function.

In G-MDSCs, CD84 induces the expression HIF1α and CMTM6, BCL2A1, and PD-L1. The transcription factor HIF1α has been implicated as a regulator of both differentiation and PD-L1 expression in these cells by directly binding hypoxia response elements in the PD-L1 promoter in MDSCs (28, 43). Stabilizing HIF1α expression decreases both proliferation and IFN secretion of T cells, and it upregulates known suppressive pathways in MDSCs (43). BCL2A1, a member of the antiapoptotic BCL2 family, has a prosurvival function. Its overexpression in G-MDSCs significantly increases the suppression of T cell proliferation and IFN-γ secretion, resulting in elevated tumor load (29).

Our study shows that MM cells induce expression of CD84 on cells in their microenvironment by secretion of the cytokine MIF. Blocking or knocking out CD84 in mice leads to a reduction in the levels of both M- and G-MDSCs, as well as a reduction in their immunosuppressive pathways, leading to an induced antitumor response by T cells and a reduction in tumor load. In addition, treating mice or human MM patient samples with anti-CD84 blocking antibody reduces the amount of MDSCs and their immunosuppression, leading to an increase in the antitumor response of T cells; this results in a reduction in the amount of tumor cells in a dose-dependent manner and increased mouse survival.

Together, these results suggest that CD84 plays a key role in regulating the function of MDSCs during MM progression, and its blockade can reduce the number of tumor cells and their immunosuppressive properties, even after the MM tumor has already been established; this renders CD84 an attractive target to treat immunosuppression, leading to an increase in the T cell–mediated antitumor response in MM.

Finally, treatment of MM-injected mice with both anti-PD1 and anti–PD-L1 has been shown to completely eliminate myeloma cells in MM mice. Initial studies of single-agent PD-1 blockade did not result in a clinical benefit. Results of the phase I/II and III trials of PD-1 blockade with ubiquitination and proteasomal degradation of Ikaros family proteins I and dexamethasone in MM had identified a patient population with potential to derive clinical benefit from this treatment approach. However, clinical trials including anti–PD-1 in combination with other treatments have been paused or halted due to serious adverse events caused by the treatment (44).

Furthermore, mice lacking PD-1 or PD-L1 spontaneously develop autoimmune diseases (45, 46), while mice lacking CD84 have been described as grossly indistinguishable from WT mice (47). Our results show that CD84 can regulate PD-1, PD-L1, and additional exhaustion markers. We therefore believe that blocking CD84 may reduce the immunosuppression that accompanies MM with fewer adverse events.

## Methods

### Mice.

C57BL/KaLwRijHsd (Harlan) CD84 deficient (CD84^–/–^) (47) mice were used at 6–8 weeks of age. The mice were age and sex matched in each experiment.

### Cells.

The 5TGM1 mouse cell line was a gift from Babatunde Oyajobi at the University of Texas Health Science Center at San Antonio, Texas, USA. The cells were cultured in 10% RPMI with 10% FCS.

### MM primary cells.

A list of patient characteristics is provided in Supplemental Table 1. Healthy BM samples are defined as samples without any evidence of abnormality, as checked BM smear and biopsy.

Human MM cell lines (MM.1S, NCI-H929, KMS-11, RPMI-8226, MM.1R and U266), and the THP1 monocytic cell line were purchased from ATCC; L363 cells were purchased from German Collection of Microorganisms and Cell Cultures. Human MM cell lines and THP1 were cultured in 10% RPMI-1640, 100 IU/mL penicillin and 100 μg/mL streptomycin.

Primary mouse stromal cells were collected from the femur and tibia by flushing out the BM, and they were seeded on 24-well plates on FCS. After 1 hour, 10% FCS RPMI medium was added, and the cells were maintained by washing of nonadherent cells and weekly changing of the 10% FCS RPMI medium.

### RNA isolation and qPCR.

Total cellular RNA from patients or mouse MDSCs was purified using the direct-zol RNA purification kit (Zymogen). cDNA was prepared using the qScript cDNA Synthesis Kit (Quantabio). qPCR was performed using SYBR green (Roche). Primers are listed in Supplemental Table 2.

Total cellular RNA from the THP1 cell line was extracted by TRIZOL reagent followed by preparation of cDNA. qPCR was performed using the TaqMan method according to the manufacturer’s instructions. The TaqMan probes for mRNA quantification were purchased from Applied Biosystems, and all reactions were performed in triplicate. OAZ1 was used as endogenous control to normalize CD84 expression.

### Staining for flow cytometry.

Cells were stained using specific antibodies as listed in Supplemental Table 3. Intracellular staining of T cells was performed as previously described (19). For intracellular staining of MDSCs, cells were stained for surface markers and then fixed with either the Fixation/Permeabilization Solution Kit (BD Bioscience) for cytoplasmic proteins or Fixation/Permeabilization Concentrate and Diluent (Thermo Fisher Scientific) for nuclear proteins.

### Western blot.

Protein lysis, separation and transfer were performed as previously described (19). Blots were probed using antibodies against β-actin (Santa Cruz Biotechnology Inc.) as a housekeeping gene, anti-CD84 (Santa Cruz Biotechnology Inc.), anti–phospho AKT (Cell Signaling Technology), anti–phospho S6 (Cell Signaling Technology), and anti–PD-L1 (Cell Signaling Technology). The bands were then detected using ECL Western Blotting Substrate.

### CD84 stimulation of human cells.

CD84 stimulation was performed as previously described (17–19).

### CD84 blocking by the B4 mAb on cells.

CD84 blocking was performed as previously described (17–19).

### MIF stimulation on human cells.

MIF stimulation was performed using CD14^+^ cells isolated from primary samples of PB with CD14 microbeads according to the manufacturer’s instructions. A total of 1 × 10^6^ CD14^+^ purified cells was incubated in RPMI medium containing 200 ng/mL of human recombinant MIF at 37°C for 18–24 hours.

Mouse BM stromal cells were first starved with 0.1% RPMI medium for 3 hours, incubated with 100 ng/mL of mouse recombinant MIF at 37°C for 48 hours, and thereafter stained for flow cytometry.

### MIF blocking by the ISO-1 MIF inhibitory on human cells.

For MIF blocking, 1 × 10^6^ isolated CD14^+^ cells were incubated for 48 hours in 1 mL 1% FCS RPMI medium containing 100 μM of the MIF antagonist ISO-1 (MilliporeSigma, 478336-92-4).

### Coculture of CD14^+^ cells with human MM cell lines..

Human PB CD14^+^ cells were isolated using CD14 microbeads (Miltenyi Biotec, catalog 130-050-201) according to the manufacturer’s instructions. Isolated CD14^+^ cells were cocultured with human myeloma cell lines for 48 hours. Following culture, CD14^+^ cells, stained with antibodies, were analyzed using flow cytometry.

### RNA-Seq.

Sorted G-MDSCs or M-MDSCs were treated with activating CD84 antibody or control, as described above, and RNA was then purified. A bulk adaptation of the MARS-Seq protocol (48) was utilized to generate the RNA-Seq libraries for expression profiling of both G-MDSCs and M-MDSCs.

CD84-activated genes in G-MDSC were classified as differentially expressed based on a cut-off of *P* < 0.05 before corrections with a base mean ≥ 5, and a log_2_ fold change of 0.58/–0.58 (1.5- or –1.5-fold of IgG values). CD84-activated M-MDSC genes that were classified as differentially expressed by the cut-off of *P* < 0.05 after *P* value corrections, with a base mean ≥ 5 and a log_2_ fold change of 0.58/–0.58 (1.5- or –1.5-fold of IgG values).

### Analysis and visualization of RNA-Seq.

Heatmap and volcano plots were prepared using the parameters stated above using R studio version 3.6.2 and the libraries dendextend, pheatmap, and ggplot2. GO analysis was conducted with R studio version 3.6.2 using the database EnrichR.

PCA and dendrogram analysis of samples was performed using Spyder version 3.3.6 Python 3.7 with the libraries numpy, matplotlib.pyplot, sklearn, and scipy.

### Chimeric mice.

Six- to 8-week-old WT or CD84^−/−^ mice were lethally irradiated with 1050 Rad. The next day, 1 × 10^6^ C57BL/KaLwRij mouse BM cells were injected into the tail vein of each mouse. After 60 days, 1 × 10^6^ 5TGM1 murine MM cells were injected into the tail vein of the WT and CD84^−/−^ mice; 3 weeks later, mice were sacrificed, and their BM, spleen, and PB were isolated.

### In vivo CD84 inhibition.

C57BL/KaLwRij mice were injected to the tail vein with 1 × 10^6^ 5TGM1 murine MM cells. After 14 days, the mice were separated into 2 or 3 groups and administered either B4 mAb or the IgG2a isotype control (BioLegend) at doses between 30 μg and 200 μg every other day into the tail vein, for a total of 5 injections or 11 injections for long-term studies.

### Virus and cellular production of CRISPR library.

The CRISPR manipulations were performed using a method similar to that from ref. 49. The following sgRNA sequences were used: control, 5′ - GAACGUAGAAAUUCCCAUUU - 3′; CD84, 5′ - GAGUCAGAAUUGUUGCUGAC - 3′.

### Chromium-51 release assay.

MM.1S and Raji cells were labeled with Chromium-51 (Cr-51) by incubating 1 × 10^6^ cells in 100 μCi of Cr-51 for 1.5 hours at 37°C. The labeled cells were washed 3 times and resuspended in complete RPMI medium, and they were plated in 96-well round-bottom plates in triplicate. Then, effector cells were added at 40:1, 20:1, and 10:1 effector/target ratios with B4 and ISO-1 or with IgG2a and DMSO controls, and they were incubated at 37°C for 16 hours. Cr-51 release was detected as previously described (50).

### Suppression assay.

MDSC suppression assay was performed as previously described (51).

### Statistics.

Analysis of data was performed using GraphPad Prism (Version 8.0, GraphPad Software Inc.). For most experiments, the mean ± SEM is provided, and at least 3 experiments were performed. For mouse experiments, due to differences in tumor load, results were normalized in each experiment to the average of the control mice, and results are therefore shown as fold change; this did not affect the percentage of immune cells, such as MDSCs, in each mouse, and these are shown as actual percentages.

To test for significance, either a 1- or 2-tailed Student’s *t* test was used depending on the experiment, unless stated otherwise (1-way ANOVA with log-rank test) Results were considered significant at *P* ≤ 0.05.

### Study approval.

All animal procedures were approved by the Animal Research Committee at the Weizmann Institute of Science. Primary patient–derived MM or healthy samples cells were obtained from City of Hope National Medical Center in compliance with an IRB-approved protocols (IRB 18067, IRB 19473); primary patient–derived MM or healthy samples cells were collected from Kaplan medical center (IRB 1339-1).

## Author contributions

HL designed research, performed experiments, analyzed data, and wrote the paper. EGG designed research, performed experiments, analyzed data, and wrote the paper. KD designed research, performed experiments, and analyzed data. LR designed research, performed experiments, and analyzed data. MPK designed research and analyzed data. MP performed experiments. BP performed experiments. JC designed research and analyzed data. TFH designed research and analyzed data. AGM designed research and analyzed data. KYT designed research and analyzed data. SB designed research and analyzed data. EC performed experiments and analyzed data. ET performed experiments. PL designed research and analyzed data. MF designed research and analyzed data. JK provided essential reagent. AK provided essential reagent. MR provided essential reagent. JY provided essential reagent. MAC provided essential reagent. OS provided essential reagent. YC provided essential reagent. SBH designed research and analyzed data. FP designed research and analyzed data. SR designed research, analyzed data, and wrote the paper. IS designed research, analyzed data, and wrote the paper.

## Supplementary Material

Supplemental data

## Version 1 01/19/2021

In-Press Preview

## Version 2 02/22/2021

Electronic publication
