# Supplementary material for: CD84 is a regulator of the immunosuppressive microenvironment in multiple myeloma
Source: JCI Insight. 2021 Feb 22;6(4):e141683. doi: 10.1172/jci.insight.141683 (PMC7934939; doi:10.1172/jci.insight.141683)
Supplement: Supplemental data [file jciinsight-6-141683-s097.pdf]

Supplementary tables

Table 1 Patient List

Patient	Age	Gender	High risk features (t(4;14), del17p, add 1q, S-phase>3%) one or more of these present	Stage of disease	State of disease (relapsed / relapsed and refractory)	Currenttth erapy	Previous transplant	Figure
1	NA	NA	NA	NA	NA	NA	NA	1A, 1G, 1H, 2H, 5M, 5Q
2	80	M	del17p	I	VGPR	RVD	no	1A, 2G, 2H, 5D, 5F, 5M, 5O, 5Q, 5A, 5B, 5C, S3A, S3C, S3E
3	84	M	No	III	VGPR	RVD	no	1A, 2H, 5M, 5Q, 5J, 5K, 5L, S3D, S3G
4	70	M	NA	Smoldering	VGPR		no	1A, 1B, 1G, 1H
5	68	M	NA	Smoldering	Stable disease	untreated	no	1A, 1B
6	71	M	NA	II	Stable disease	Vel-dex	no	1A
7	94	M	NA	III	VGPR	RVD	no	1A, 3D, 3E, 3F, 3G, 3J, 3K
8	78	F	Myeloma not proved	Smoldering	Stable disease	untreated	no	1A, 1B, 3E, 3F, 3G, 3K
9	84	M	NA	III	PR	RVD	no	1A, 1G, 1H, 2H, 3D, 3E, 3G, 3H, 3I, 3J, 3K, 5E, 5G, 5M, 5N, 5O, 5P, 5Q, 5R, S3A, S3B
10				MGUS				1A
11	85	M	Hyperdiploid (low risk)	I	VGPR	Vel-Dex	no	1A, 2B
12	82	M	NA	II	Refractory	Vel-Dex	no	1A, 1B, 2A
13	NA	NA	NA	NA	NA	NA	NA	
14	79	F	del P53 ,gain 1q21, del 1p32, t(11;14),del 13q	PLASMA CELL LEUKEMIA	Refractory	VCD	no	1A, 1B, 2A
15				MGUS				1A
16	64	F	1 q gain	Smoldering	Stable disease	untreated	no	1A, 1G, 1H, 2H, 5M, 5O, 5J, 5K, 5L, S3D, S3G, 5A, 5B, 5C,

								S3A, S3C, S3E
17	65	F	NA	III	VGPR	VCD	yes	1A, 5D, 5E, 5O, 5Q, 5J, 5K, 5L, S3D, S3G, 5A, 5B, 5C, S3A, S3C, S3E
18	79	F	NA	PCL	refractory	Dexa	no	1A, 1G, 1H, 2G, 2H, 5D, 5E, 5M, 5A, 5B, 5C, S3A, S3C, S3E
19	NA	NA	NA	NA	NA	NA	NA	1B
20	NA	NA	NA	NA	NA	NA	NA	1B, 2B
21	82	F	NA	II	Partial response	VMP	no	1B, 2B
22	60	M	1q21 gain, del 13q, t(4:14 translocation)	II	PR	VCD		1B, 2A
23	84	F	NA	II	refractory	Vel-Dex	no	2A
24	60	F	NA	II			no	2A, 2B
25	73	M	NA	III	refractory	Vel-Dex	no	3D, 3E, 3H, 3I, 3J, 3K, 5E, 5G, 5N, 5P, 5R
26	NA	NA	NA	NA	NA	NA	NA	1A, IG, 3D, 3E, 3H, 3I, 3J, 3K
27	NA	NA	NA	Plasma cell leukemia	NA	NA	NA	1A, 1G, 1H, 3D, 3E, 3F, 3G, 3H, 3I, 3J, 3K
28	NA	NA	NA	NA	NA	NA	NA	3D, 3E, 3F, 3G, 3H, 3J, 3K
29	70	F	t(14:16)<br>1q21+<br>Del13q	ISS I	Newly diagnosed	no	no	1A, 1G, 1H, 5E, 5G, 5N, 5P, 5R
30	82	F	1q21+<br>Del13q	ISS II	Newly diagnosed	no	no	1A, 1G, 1H, 3D, 3E, 3F, 3G, 3H, 5E, 5G
31	75	F	1q21+<br>hyperdiploid	Not done	Partial response	Thal+ Cycloph<br><br>VCD<br>RD	2009<br>2014	3D, 3E, 3G, 3I, 3K, 5E, 5G, 5N, 5P, 5R
32	68	M	Del17p<br>1q21+<br>Del13q	ISS III	VGPR	PACE<br>VCD	01/19<br>05/19	3D, 3E, 3G, 3K, 5E, 5G,

			T(14:16)<br>1p32-			Velcade maintenance		5N, 5P, 5R
26	NA	NA	NA	NA	NA	NA	NA	1A, IG, 3D, 3E, 3H, 3I, 3J, 3K
27	NA	NA	NA	Plasma cell leukemia	NA	NA	NA	1A, 1G, 1H, 3D, 3E, 3F, 3G, 3H, 3I, 3J, 3K
28	NA	NA	NA	NA	NA	NA	NA	3D, 3E, 3F, 3G, 3H, 3J, 3K
29	70	F	t(14:16)<br>1q21+<br>Del13q	ISS I	Newly diagnosed	no	no	1A, 1G, 1H, 5E, 5G, 5N, 5P, 5R
30	82	F	1q21+<br>Del13q	ISS II	Newly diagnosed	no	no	1A, 1G, 1H, 3D, 3E, 3F, 3G, 3H, 5E, 5G
31	75	F	1q21+<br>hyperdiploid	Not done	Partial response	Thal+<br>Cycloph<br><br>VCD<br>RD	2009<br>2014	3D, 3E, 3G, 3I, 3K, 5E, 5G, 5N, 5P, 5R
32	68	M	Del17p<br>1q21+<br>Del13q<br>T(14:16)<br>1p32-	ISS III	VGPR	PACE<br>VCD<br>Velcade maintenance	01/19<br>05/19	3D, 3E, 3G, 3K, 5E, 5G, 5N, 5P, 5R

Patients #24 and pat # 17 are the same patient in different time points – diagnosis and relapse (the earlier is at diagnosis). At diagnosis she reponded to treatment. At relapse she was refractory

NA= not available
VGPR= very good partial response
RVD = Revlimide + Velcade + Dexamethasone
DD= Daratumumab + Dexamethasone

Table 2 Real-Time Primers

target	species	forward primer	reverse primer
HIF1a	human	GAACGTCGAAAAGAAAAGTCTCG	CCTTATCAAGATGCGAACTCAC
CD84	human	TTGTTCCGTTTGTTCAAGAG	CGGAATAAACTGTGTTCACTG
S100A9	human	GTCGCAGCTGGAACGCAACA	CCTGGCCTCCTGATTAGTGG
CYBA	human	ATGGGGCAGATCGAGTGGGCCAT	GTAGATGCCGCTCGCAATGGCCAA
CMTM6	human	ATGAAGGCCAGCAGAGACAG	GTGTACAGCCCCACTACGGA
BCL2A1	human	GTCATCCAGCCAGATTTAGGTTC	CCCGGATGTGGATACCTATAAGG
PSMB2	human	AGGTTGGCAGATTCAGGATG	AGAGGGCAGTGGAACTCCTT
PD-L1	human	TGGCATTTGCTGAACGCATTT	AGTGCAGCCAGGTCTAATTGT
Bcl2	mouse	GCTACCGTCGTGACTT	GCCGGTTCAGGTACTC
Rpl32	mouse	TTAAGCGAAACTGGCGGGAAAC	TTGTTGCTCCCATAACCGATG
CD84	mouse	ATATAGCTGGAGTCCCTTTGGAG	AAAGAGCACGGCCAATCCTC
PD-L1	mouse	GAGCTGATCATCCCAGAACTGC	GACCGTGGACACTACAATGAGGA
CMTM6	mouse	ATGGAGAACGGAGCGGTCTA	CACACTCGGACACAACCTCT
BCL2A1	mouse	TGGAAACTTGTTTGTAAGCACGT	CTTCAGTATGTGGCTACAGGTACC
CYBA	mouse	AGATCGAGTGGGCCATGT	ACCACTGTGTGAAACGTCCA
S100A9	mouse	CACAGTTGGCAACCTTTATG	CAGCTGATTGTCCTGGTTTG

Table 3 List of antibodies used

Target	Species	Fluorophore	Company	Clone
CD138	Mouse	APC	BD bioscience	281-2
PD-L1	Human	PE/CY7	Biolegend	29E.2A3
PD-L1	Mouse	BV711	Biolegend	10F.9G2
B220/CD45R	Human/Mouse	FITC or APC	Biolegend	RA3-6B2
CD38	Human	FITC/PE	Biolegend	HIT2
CD19	Human	APC-CY7	Biolegend	HIB19
CD3	Mouse	APC-CY7	Biolegend	145-2C11
CD3	Human	Pacific blue	Biolegend	UCHT1
CD4	Mouse	FITC/PerCP-Cyanine5.5	Biolegend/BioGems	RMA4-5/GK1.5
CD4	Human	FITC	Biolegend	OKT4
CD8	Mouse	PE	Biolegend	53-6.7
CD8	Human	APC-CY7	Biolegend	HIT8a
PD1	Mouse	PE/CY7	Biolegend	29F.1A12
PD1	Human	BV711	BD Biosciences	EH12.1
Lag-3	Mouse	PE	BD Biosciences	C9B7W
Lag-3	Human	APC	Biolegend	11C3C65
CTLA-4	Mouse	APC	Biolegend	UC10-4B9
CTLA-4	Human	PE-CY7	Biolegend	L3D10
2B4	Mouse	FITC	Biolegend	m2B4.(B6)45B.1
KLRG-1	Mouse	Efluor405	eBioescience	KLRG1
LAMP-1	Mouse	PE	Biolegend	1D48
Granzyme B	Mouse/Human	APC	Biolegend	GB11
IL-2	Mouse	PE-CY7	Biolegend	JES6-5H4
IFNgamma	Mouse	Pacific blue	Biolegend	XMG1.2
S100A9	Mouse/Human	APC	ThermoFisher Scientific	MAC387
CMTM-6	Mouse/Human	unconjugated	St John's Laboratory	
CYBA	Mouse/Human	unconjugated	St John's Laboratory	
BCL2A1	Mouse/Human	unconjugated	St John's Laboratory	
HIF1α	Mouse/Human	PE	RnD Systems	241812
Goat Anti-Rabbit IgG, Fc Fragment Specific	Anti-Rabbit	Alexa fluor 647	Jackson ImmunoResearch	

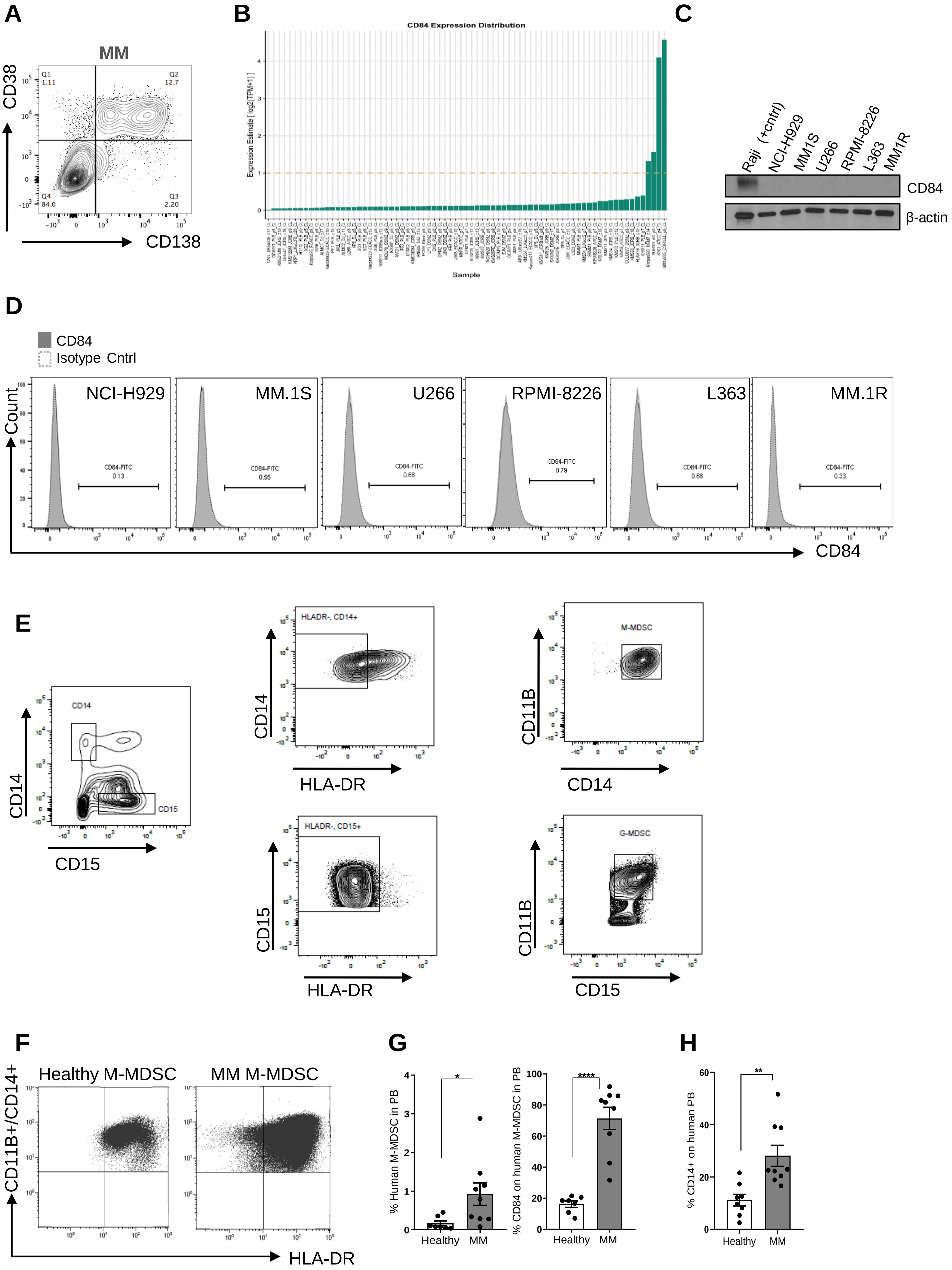

Figure S1 - ***CD84 expression in MM and MDSCs***

(A) Gating strategy for CD38+CD138+ primary human MM cells. **(B)** mRNA levels of CD84 in various MM cell lines. Data was taken from CoMMpass IA9 dataset. **(C)** CD84 protein expression was determined on six human MM cell lines by western blot analysis. **(D)** CD84 expression on six human MM cell lines analyzed by flow cytometry. **(E)** Gating strategy for G- and M- MDSCs. **(F)** Gating of $CD14^+$, $CD11B^+$, $CD15^-$, $HLA\text{-}DR^-$ M-MDSCs in PB derived from Healthy or MM patients. Representative dot plots are shown on the left (n=7-9, ****p<0.0001). (**G**) Percentage of M-MDSCs and CD84 expression on M-MDSCs in PB and BM derived from Healthy or MM patients (n=5-9, **p<0.01). (**H**) Percentage of CD14+ populations in PB derived from Healthy or MM patients (n=5-9, **p<0.01).

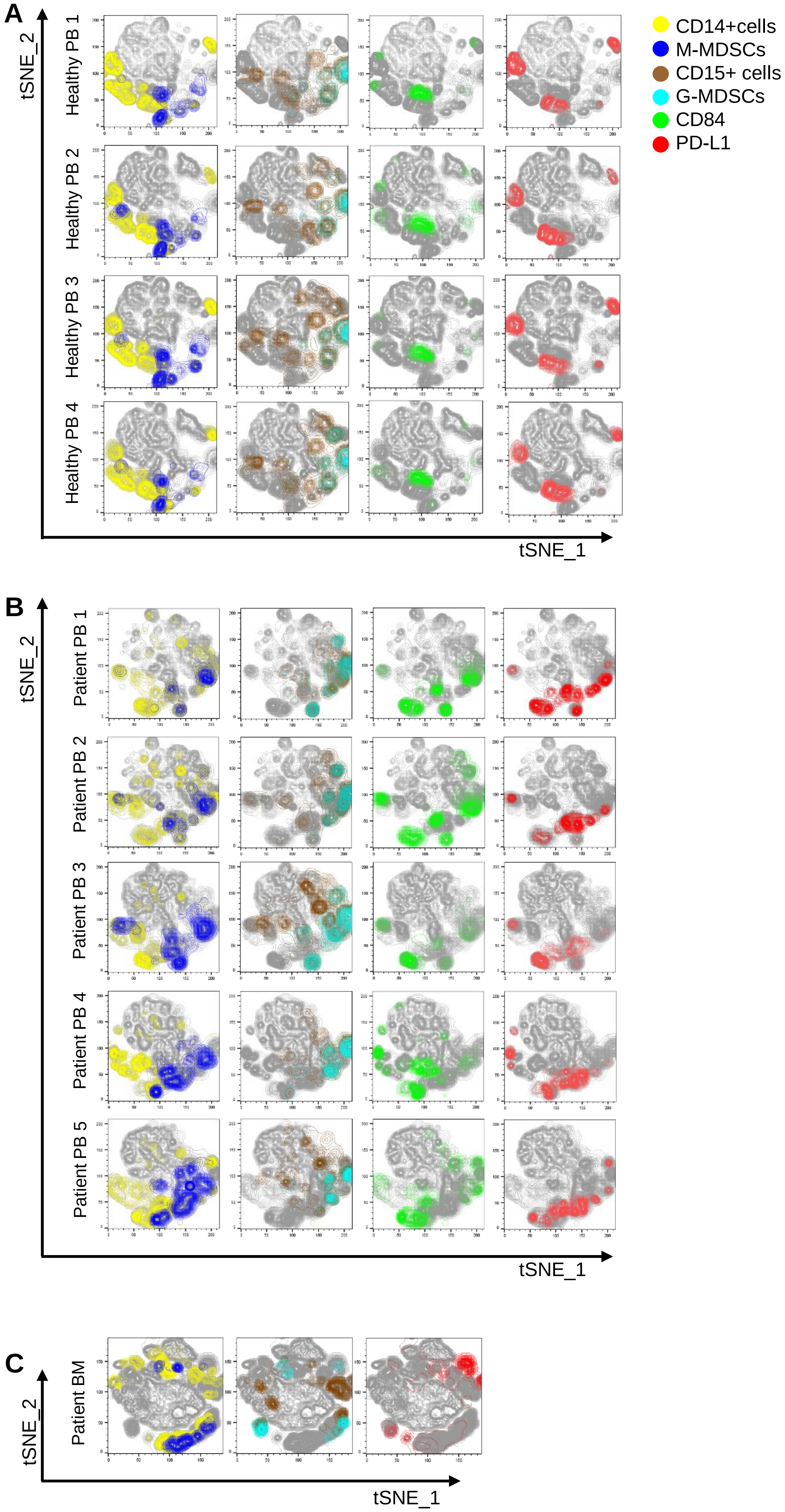

Figure S2 - ***t-SNE plots of MDSCs from healthy and MM donors***

(A-B) t-SNE plots based on CD14+, CD15+, M-MDSC, G-MDSC, CD84+ and PD-L1+ cells from PB of healthy donors (A), and MM patients (B). **(C)** Representative t-SNE plots based on CD14+, CD15+, M-MDSC, G-MDSC, and PD-L1 + cells from BM derived from MM patient samples. t-SNE was run with no PCA step, perplexity = 30, 1000 iterations. From each of the samples, 200, 000 cells were randomly selected. Cells are colored according to the expression level of CD14+/CD15- for CD14; CD14+/CD15-/CD11b+/HLA-DR low/- for M-MDSC; CD15+/CD14- for CD15; and CD15+/CD14-/CD11b+/HLA-DR low/- for G-MDSC, CD84, and PD-L1 markers.

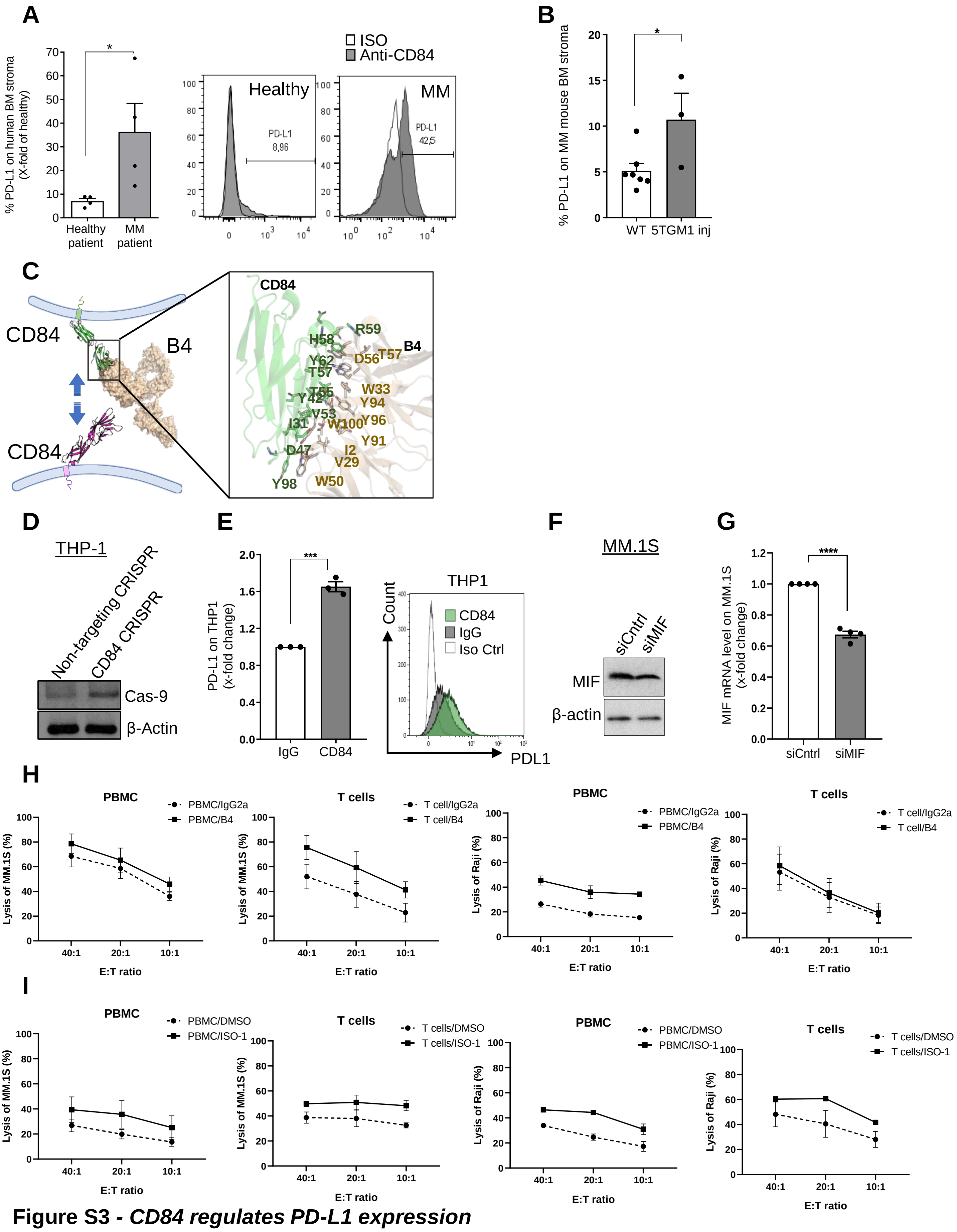

Figure S3 - *CD84 regulates PD-L1 expression*

(A) Bone marrow aspirates from healthy donors or MM patients were grown in culture. PD-L1 cell surface levels were analyzed by FACS. Representative histograms are shown on the right (n=4, *p<0.05). **(B)** PD-L1 expression on stroma grown from BM of 5TGM1 injected C57BL/KaLwRij WT mice (n=3-6, *p<0.05). **(C)** The model for B4 binding to CD84. The Schrodinger Antibody Modeling Interface was used to predict the structure of the B4 antibody, ZDOCK for the prediction of antibody-antigen complex, and Prime-MMGBSA for optimization and calculation of binding free energy. The complex with the lowest binding free energy was selected. The predicted structure shows a binding free energy of -100 kcal/mol, indicative of strong binding. **(D)** LentiCRISPR-v2 CRISPR/Cas9 system was established in THP1 cell line with constitutive knockout of CD84 expression, the expression of Cas9 was determined by western blot analysis. **(E)** PD-L1 expression level on THP1 cells upon 48 hrs stimulation with an agonistic anti-CD84 (n=3, ***p<0.001). **(F-G)** MM.1S cells were transfected with MIF or control siRNAs, 48hrs. The transfected cells were analyzed for MIF protein and mRNA levels with WB and qPCR, respectively n=4, *p<0.05, paired t-test. **(H-I)** Cr-51 release assay for killing of MM.1S and Raji cells. Cr-51 labelled MM.1S and Raji cells (control cell line) were incubated with effector cells in 40:1, 20:1 and 10:1 effector-to-target ratios with B4 and ISO-1 or IgG2a and DMSO controls for 16 hrs at 37°C. Supernatants were collected and the released Cr-51 was counted using a gamma counter. Results represent the mean from three independent experiments.

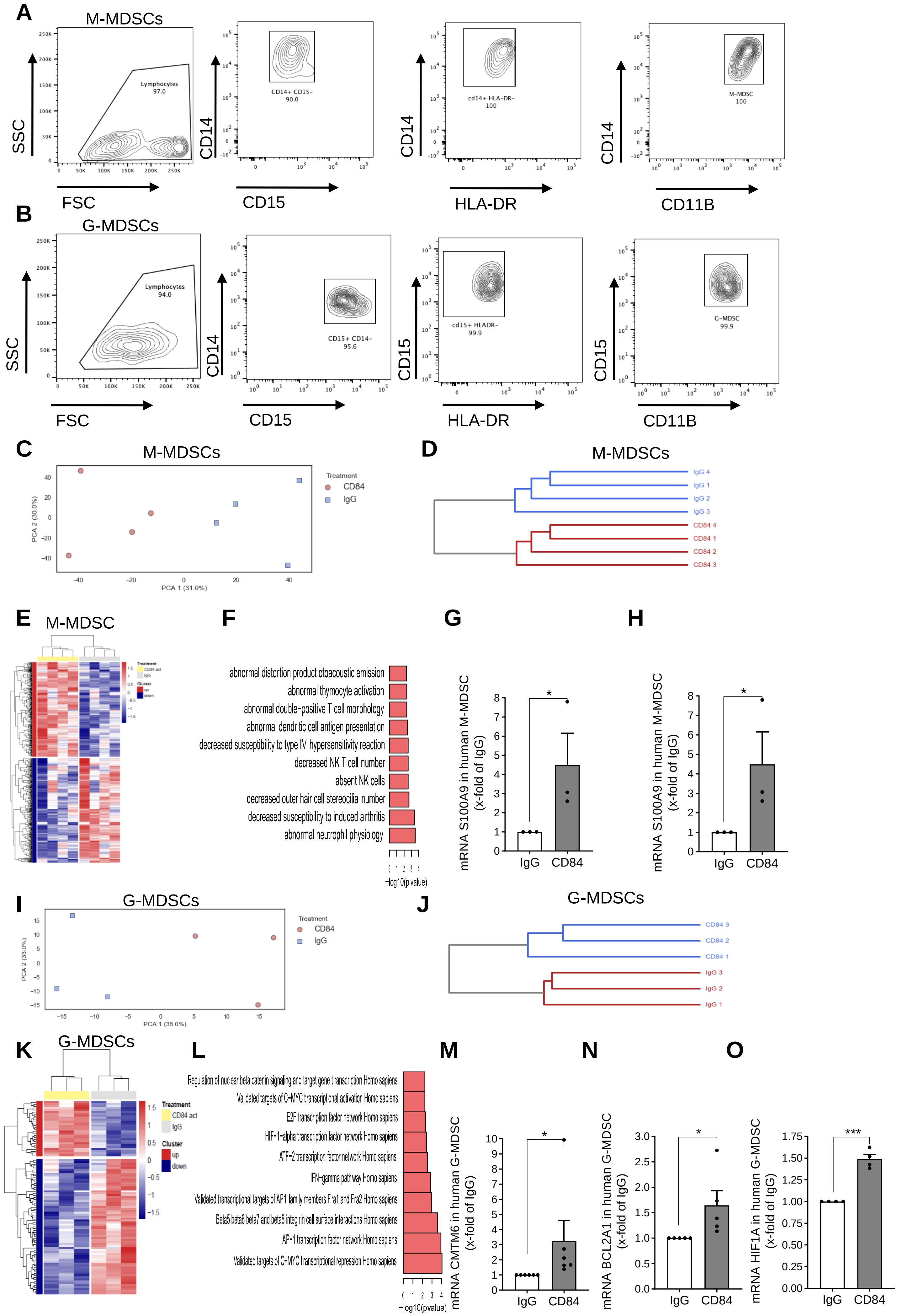

Figure S4 - *CD84 induced cascades in MDSCs*

(A-B) M-MDSCs ($CD14^+$, $CD15^-$, HLA-DR^-, $CD11B^+$) (A), or G-MDSCs ($CD15^+$, $CD14^-$, HLA-DR^-, $CD11B^+$) (B) derived from BM aspirates were sorted. Dot plots show the sorted populations. **(C)** Principal component analysis of M-MDSCs following RNA-seq. **(D)** Dendrogram of M-MDSCs following RNA-seq. **(E)** Heatmap of all differentially expressed genes from M-MDSC RNA-seq. **(F)** Highlighted gene ontology terms from the upregulated DE genes of M-MDSCs (MGI Mammalian Phenotype 2019, Enriche R). **(G-H)** S100A9 (G) and CYBA (H) mRNA expression by qPCR (n=3, *$p<0.05$, paired t-test). **(I)** Principal component analysis of G-MDSCs following RNA-seq. **(J)** Dendrogram of G-MDSCs following RNA-seq. **(K)** Heatmap of all differentially expressed genes from G-MDSCs RNA-seq. **(L)** Highlighted gene ontology terms from the upregulated DE genes of G-MDSCs (NCI Nature 2016, Enriche R). **(M-O)** CMTM6 (M), BCL2A1 (N) and HIF1A (O) mRNA levels were determined by qRT-PCR (n=6, *$p<0.05$, ***$p<0.001$, paired t-test).

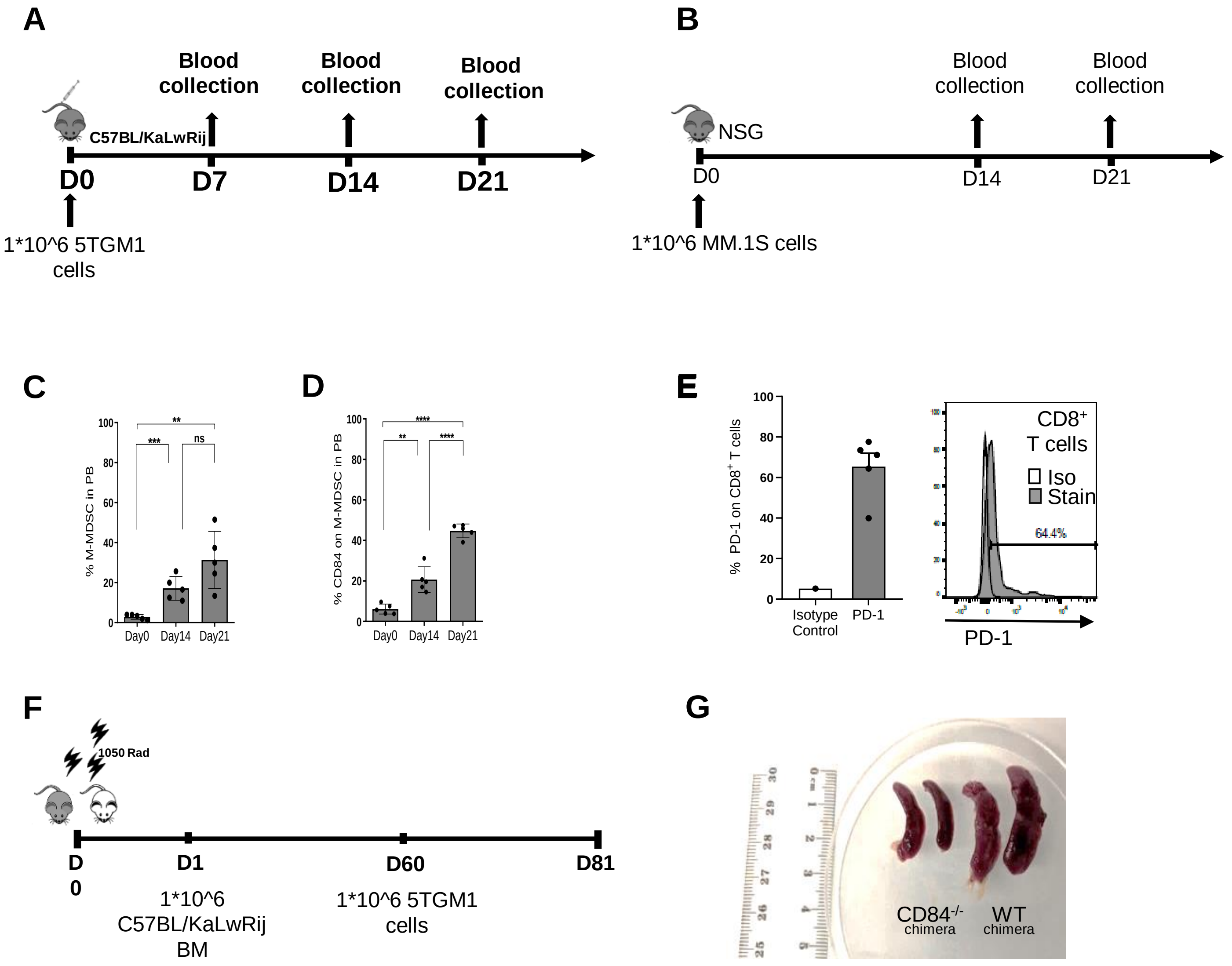

Figure S5 ***- The in vivo role of CD84***

(A) A-C) Schematic depiction of the experimental protocol for 5TGM1 cells I.V. injected into syngeneic immune-competent C57BL/KaLwRij mice. PB samples were collected and analysed after 1 week, 2 weeks, and 3 weeks by weekly submandibular bleeding after the injection. **(B-D)** MM.1S cells were injected by I.V. into immuno-deficient NSG mice. After MM cell engraftment, peripheral blood from the mice were collected by submandibular bleeding once a week. Experimental scheme of the study (B), PB M-MDSC percentage (C) and CD84 expression on these cells (D) by FACS (n=5, M-MDSC **p<0.01, n=5, CD84+ M-MDSC, ****p < 0.0001). **(E)** PD-1 expression was analyzed on bone marrow CD8+ T cells by FACS (n=5). **(F-G)** WT or CD84-/- mice were irradiated with 1050 Rad and injected with 1x106 C57BL/KaLwRij BM cells after 1 day. After 60 days, the mice were injected with 1x10^{6} 5TGM1 cells. After an additional 21 days, the mice were sacrificed, and their bone marrow, blood and spleens analysed. (F) The experimental model. (G) Photograph of WT and CD84 deficient spleens.

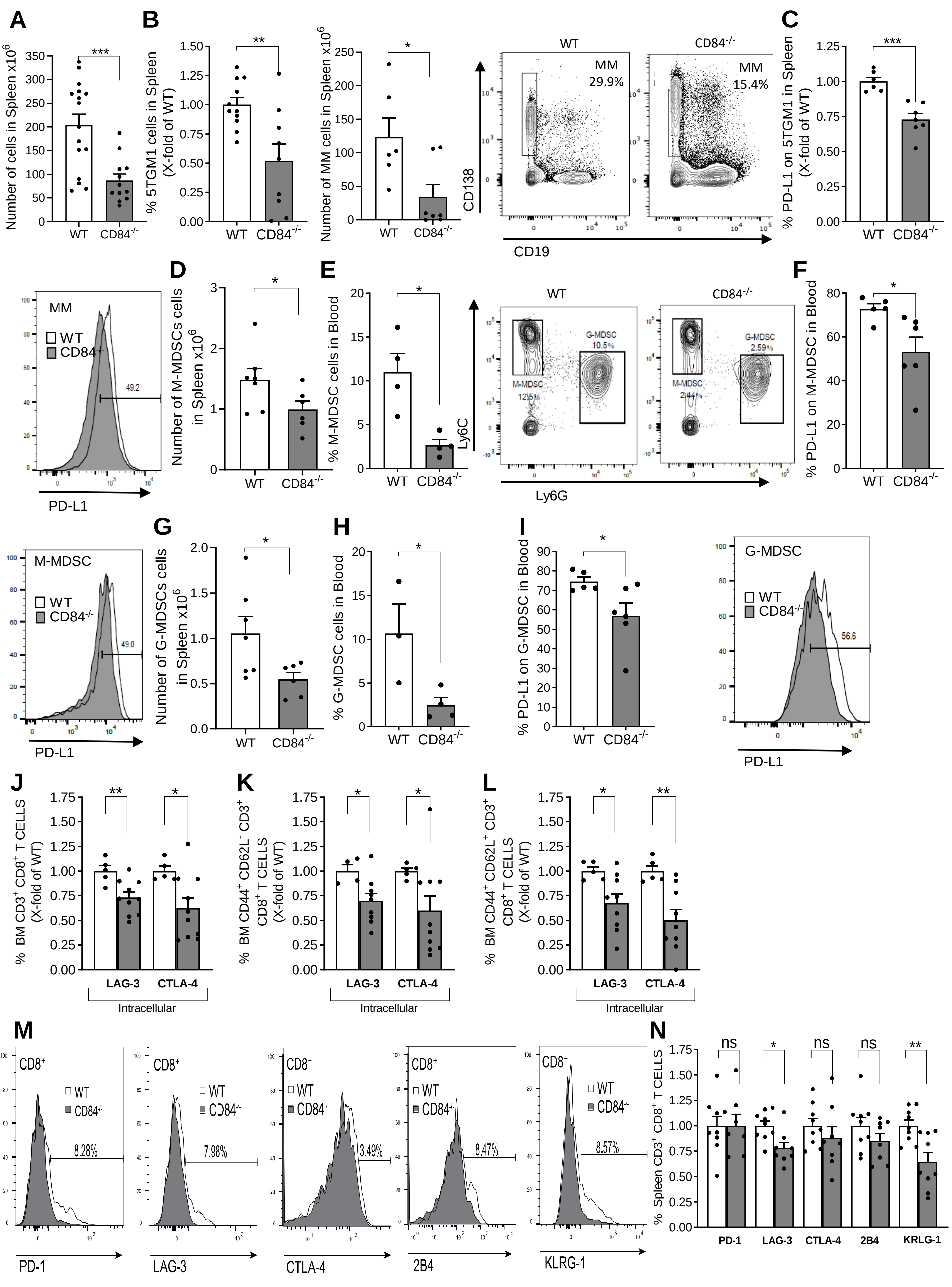

Figure S6- CD84 deficiency reduces tumor load, MDSCs function and induce T cell activity

CD84$^{-/-}$ mice backcrossed to C57BL/KaLwRij for 6-7 generations and C57BL/KaLwRij mice were injected with 5TGM1 cells. After 28 days, the mice were sacrificed and their bone marrow, blood and spleens were analyzed. **(A)** Cell numbers in spleens (n=12-16, ***p<0.001). **(B)** Percentage and number of MM cells in the spleen. Representative dot plots shown on the right (n=6-11, **p<0.01). **(C)** Percentage of PD-L1 on MM cells in the spleen. Representative dot plots shown on the right (n=6, ***p<0.001). **(D)** Number of M-MDSCs in spleen (n=6-7, *p<0.0.5, one-tailed T-test). **(E)** Percent of M-MDSCs in peripheral blood. Representative dot plot are shown in the right (n=5-6, *p<0.05). **(F)** PD-L1 expression levels on M-MDSCs derived from peripheral blood. Representative histogram shown on the right (n=4, *p<0.05). **(G)** Percent of G-MDSCs in peripheral blood. (n=3-4, *p<0.05). **(H)** Number of G-MDSCs in spleen (n=6-7, *p<0.0.5). **(I)** PD-L1 levels on PB G-MDSCs in peripheral blood with representative histogram shown (n=5-6, *p<0.05). **(J-L)** BM CD8^{+} T cells were analyzed for LAG-3 and CTLA-4 intracellularly in all T cells (K), in the effector memory subset (L) and central memory subset (M) n=4-10, *p<0.05, **p<0.01). **M)** Representative plots for BM derived T cells analyzed for PD-1, LAG-3, CTLA-4, 2B4 and KLRG-1. **(N)** Splenic T cells were analyzed for PD-1, LAG-3, CTLA-4, 2B4 and KLRG-1 (n=7-9, ns>0.05, *p<0.05, **p<0.01).

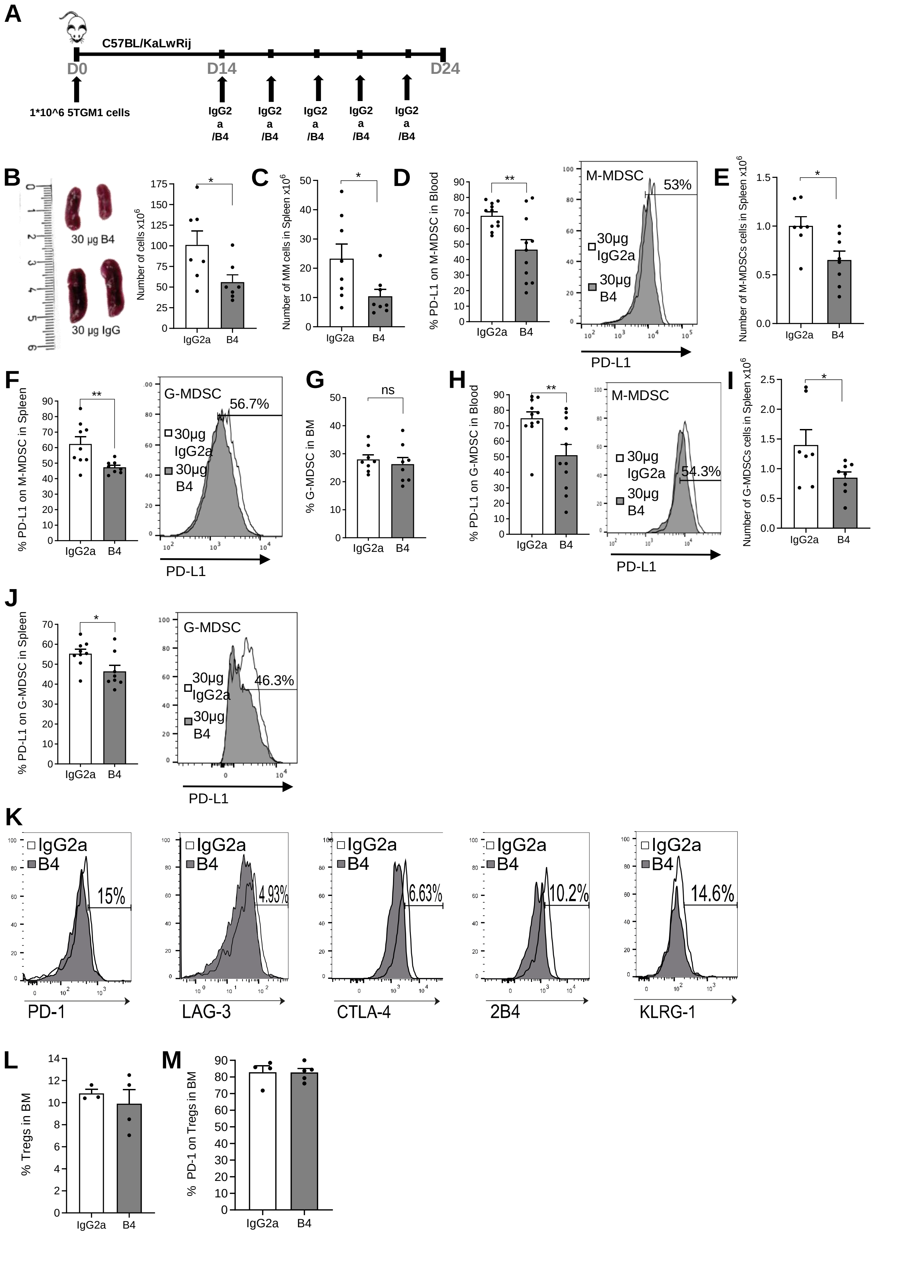

Figure S7- B4 has a therapeutic effect on MM induced mice.

(A-M) C57BL/KaLwRij mice were injected with $1x10^6$ 5TGM1 cells. After 2 weeks, the mice were divided into two groups and treated either with five injections of 30 mg anti-CD84 blocking antibody, B4, or IgG2a control for five injections. (A) The experimental settings. (B) Pictures of spleens derived from B4 or IgG2a treated mice and cell numbers (n=7, *p<0.05). (C) Number of MM cells in the spleen (n=8, *p<0.05). (D) Percent of PD-L1 protein levels on PB M-MDSCs cells in the blood (n=11, **p>0.01). (E) Number of M-MDSCs cells in the spleen (n=7-8, *p<0.05). (F) Percent of PD-L1 on M-MDSCs cells in the spleen (n=8-9, **p>0.01). (G) Percent of G-MDSCs cells in the bone marrow (n=8, p>0.05). (H) Percentage of PD-L1 expressed on PB G-MDSCs (n=11, **p>0.01). (I) Number of G-MDSCs cells in the spleen (n=7-8, *p<0.05). (J) Percentage of PD-L1 expressed splenic G-MDSCs (n=11, **p>0.01). (K) PD-1, LAG-3, CTLA-4, 2B4 and KLRG-1 were analyzed on bone marrow-derived T cells by FACS. Representative histograms are provided. (L) Percent of Tregs in the bone marrow (n=3-4, p=0.58). (M) Percentage of PD-1 on Tregs in the bone marrow (n=4, p=0.97).

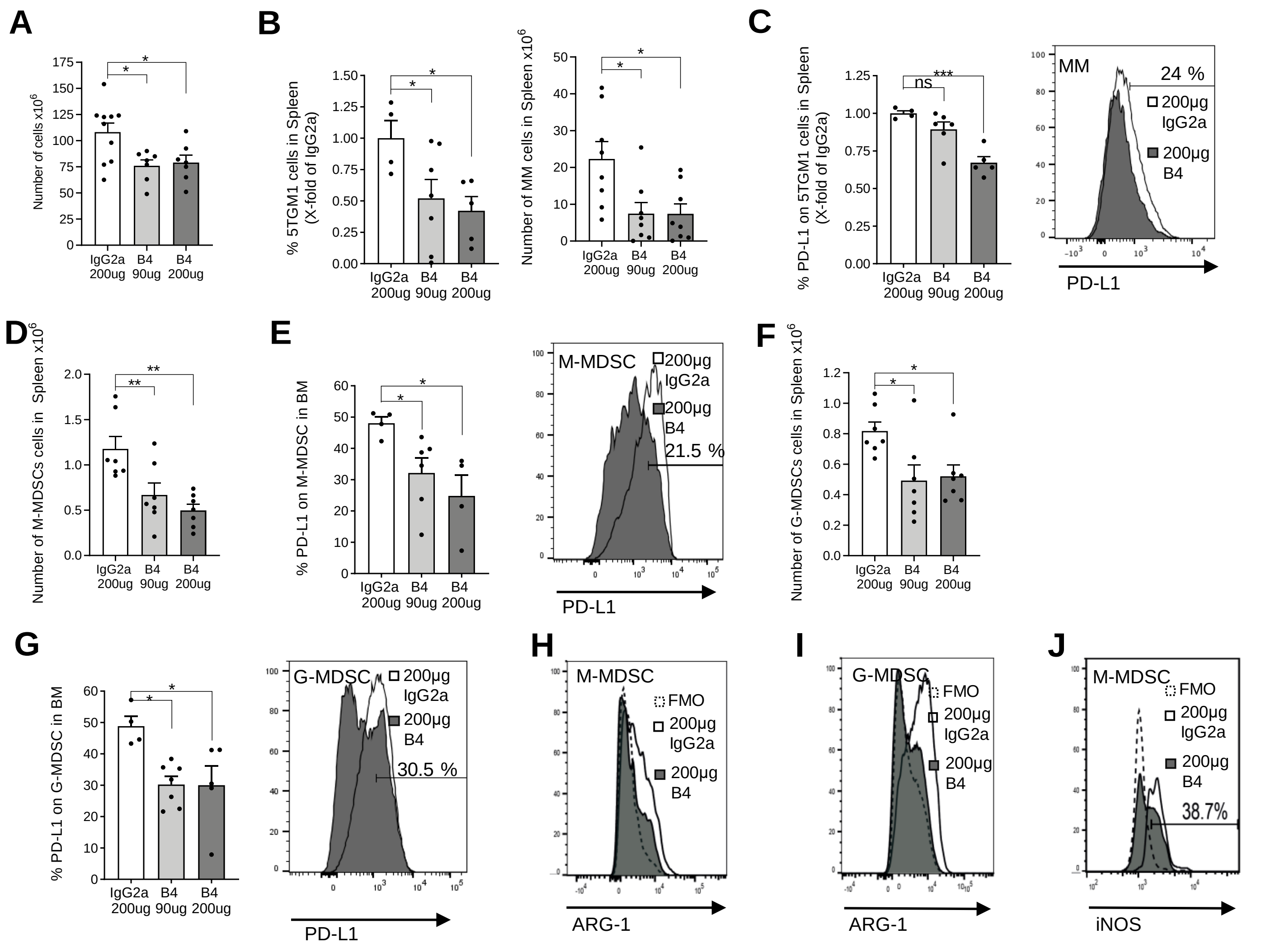

Figure S8 - B4 has a dose-dependent therapeutic effect on MM induced mice.

(A-J) C57BL/KaLwRij mice were injected with $1x10^6$ 5TGM1 cells. After 2 weeks, the mice were divided into three groups and treated five times with 90 µg or 200 µg of B4, or 200µg IgG2a. (A) Spleen cell numbers (n=7, *p<0.05) (B) Percent and number of MM cells in the spleen (n=4-7, *p<0.05, one-way ANOVA with Holm-Sidak multiple corrections test). (C) Percent of PD-L1 on MM cells in the spleen. Representative histograms are shown on the right (n=4-6, ***p<0.001, one-way ANOVA with Holm-Sidak multiple corrections test). (D) Number of M-MDSC cells in the spleen (n=7-8, **p<0.01, one-way ANOVA with Holm-Sidak multiple corrections test). (E) Percentage of PD-L1 on BM M-MDSCs. Representative histograms shown on the right (n=4-6, *p<0.05, one-way ANOVA with Newman-Keuls corrections test). (F) Number of G-MDSC cells in the spleen (n=7-8, **p<0.01, one-way ANOVA with Holm-Sidak multiple corrections test). (G) Percent of PD-L1 on BM G-MDSCs. Representative histograms are shown on the right (n=4-7, *p<0.05, one-way ANOVA with Holm-Sidak multiple corrections test). (H-J) ARG-1 (H-I) and iNOS (J) were analyzed on bone marrow-derived MDSCs by FACS. Representative histograms are provided.
